# Shared differential factors underlying individual spontaneous neural activity abnormalities in major depressive disorder

**DOI:** 10.1017/S0033291724002617

**Published:** 2024-11

**Authors:** Shaoqiang Han, Ya Tian, Ruiping Zheng, Baohong Wen, Liang Liu, Hao Liu, Yarui Wei, Huafu Chen, Zongya Zhao, Mingrui Xia, Xiaoyi Sun, Xiaoqin Wang, Dongtao Wei, Bangshan Liu, Chu-Chung Huang, Yanting Zheng, Yankun Wu, Taolin Chen, Yuqi Cheng, Xiufeng Xu, Qiyong Gong, Tianmei Si, Shijun Qiu, Ching-Po Lin, Yanqing Tang, Fei Wang, Jiang Qiu, Peng Xie, Lingjiang Li, Yong He, Yuan Chen, Yong Zhang, DIDA-MDD Working Group, Jingliang Cheng

**Affiliations:** 1 Department of Magnetic Resonance Imaging, The First Affiliated Hospital of Zhengzhou University, Zhengzhou, Henan Province, China; 2 The Clinical Hospital of Chengdu Brain Science Institute, School of Life Science and Technology, University of Electronic Science and Technology of China, Chengdu, China; 3 School of Medical Engineering, Xinxiang Medical University, Xinxiang, Henan Province, China; 4 State Key Laboratory of Cognitive Neuroscience and Learning, Beijing Normal University, Beijing, China; 5 Beijing Key Laboratory of Brain Imaging and Connectomics, Beijing Normal University, Beijing, China; 6 IDG/McGovern Institute for Brain Research, Beijing Normal University, Beijing, China; 7 School of Systems Science, Beijing Normal University, Beijing, China; 8 Key Laboratory of Cognition and Personality (SWU), Ministry of Education, Chongqing, China; 9 Department of Psychology, Southwest University, Chongqing, China; 10 Department of Psychiatry, National Clinical Research Center for Mental Disorders, The Second Xiangya Hospital of Central South University, Changsha, Hunan, China; 11 Mental Health Institute of Central South University, China National Technology Institute on Mental Disorders, Hunan Key Laboratory of Psychiatry and Mental Health, Hunan Medical Center for Mental Health, Changsha, Hunan, China; 12 Key Laboratory of Brain Functional Genomics (Ministry of Education), Affiliated Mental Health Center (ECNU), School of Psychology and Cognitive Science, East China Normal University, Shanghai, China; 13 Department of Radiology, The First Affiliated Hospital of Guangzhou University of Chinese Medicine, Guangzhou, China; 14 Peking University Sixth Hospital, Peking University Institute of Mental Health, NHC Key Laboratory of Mental Health (Peking University), National Clinical Research Center for Mental Disorders (Peking University Sixth Hospital), Peking University, Beijing, China; 15 Huaxi MR Research Center (HMRRC), Department of Radiology, West China Hospital, Sichuan University, Chengdu, China; 16 Department of Psychiatry, First Affiliated Hospital of Kunming Medical University, Kunming, China; 17 Research Unit of Psychoradiology, Chinese Academy of Medical Sciences, Chengdu, Sichuan, China; 18 Institute of Neuroscience, National Yang Ming Chiao Tung University, Taipei, Taiwan; 19 Department of Education and Research, Taipei City Hospital, Taipei, Taiwan; 20 Department of Psychiatry, The First Affiliated Hospital of China Medical University, Shenyang, China; 21 Chongqing Key Laboratory of Neurobiology, Chongqing, China; 22 Department of Neurology, The First Affiliated Hospital of Chongqing Medical University, Chongqing, China; 23 Chinese Institute for Brain Research, Beijing, China

**Keywords:** amplitude of low-frequency fluctuations, dimension, heterogeneity, major depressive disorder, normative modeling

## Abstract

**Background:**

In contemporary neuroimaging studies, it has been observed that patients with major depressive disorder (MDD) exhibit aberrant spontaneous neural activity, commonly quantified through the amplitude of low-frequency fluctuations (ALFF). However, the substantial individual heterogeneity among patients poses a challenge to reaching a unified conclusion.

**Methods:**

To address this variability, our study adopts a novel framework to parse individualized ALFF abnormalities. We hypothesize that individualized ALFF abnormalities can be portrayed as a unique linear combination of shared differential factors. Our study involved two large multi-center datasets, comprising 2424 patients with MDD and 2183 healthy controls. In patients, individualized ALFF abnormalities were derived through normative modeling and further deconstructed into differential factors using non-negative matrix factorization.

**Results:**

Two positive and two negative factors were identified. These factors were closely linked to clinical characteristics and explained group-level ALFF abnormalities in the two datasets. Moreover, these factors exhibited distinct associations with the distribution of neurotransmitter receptors/transporters, transcriptional profiles of inflammation-related genes, and connectome-informed epicenters, underscoring their neurobiological relevance. Additionally, factor compositions facilitated the identification of four distinct depressive subtypes, each characterized by unique abnormal ALFF patterns and clinical features. Importantly, these findings were successfully replicated in another dataset with different acquisition equipment, protocols, preprocessing strategies, and medication statuses, validating their robustness and generalizability.

**Conclusions:**

This research identifies shared differential factors underlying individual spontaneous neural activity abnormalities in MDD and contributes novel insights into the heterogeneity of spontaneous neural activity abnormalities in MDD.

## Introduction

Major depressive disorder (MDD) stands out as a widespread and debilitating psychiatric condition, holding the dubious distinction of being the foremost cause of global disability (Winter et al., 2022). The heterogeneity within MDD is striking, with patients exhibiting a diverse array of symptoms to the extent that two cases can present markedly different symptom profiles as outlined in the Diagnostic and Statistical Manual of Mental Disorders, 5th edition criteria for MDD (Goldberg, 2011). This heterogeneity extends beyond symptoms, encompassing variations in etiologies, responses to treatment, and the trajectories of the disease (Drysdale et al., 2017; Shelton, 2007). Despite this pronounced diversity, most neuroimaging studies still rely on traditional case-control approaches, geared toward uncovering group-level effects. However, it is increasingly evident that such approaches only capture effects of a fraction of patients and mask the distinct neuroimaging characteristics unique to individuals (Lv et al., 2021; Marquand, Rezek, Buitelaar, & Beckmann, 2016; Wolfers et al., 2018). The heterogeneity among cases is one of the leading causes of inconsistent neuroimaging findings in these researchers (Chen et al., 2016). It is urgent to develop approaches to identifying person-specific neuroimaging differential patterns to facilitate precision clinical decision-making.

Magnetic resonance imaging (MRI) studies have uncovered anomalies in both brain structure and intrinsic activity widespread brain regions in MDD (Gong et al., 2020; Ho et al., 2022; Schmaal et al., 2017). Nevertheless, the consistency of findings poses a persistent challenge (Chen et al., 2016). While discrepancies can be attributed, in part, to variations in scanning parameters, analysis pipelines, and small sample sizes (Schmaal et al., 2017), growing evidence suggests that psychiatric disorders, including MDD, are highly heterogeneous syndromes (Buch & Liston, 2021; Lynch, Gunning, & Liston, 2020; Wen et al., 2022). To address the heterogeneity, researchers typically identify more homogeneous subtypes based on neuroimaging, clinical characteristics, or a combination of both (Beijers, Wardenaar, van Loo, & Schoevers, 2019; Lynch et al., 2020; van Hulst, de Zeeuw, & Durston, 2015). Some have gone a step further, using normative modeling to reveal individual-specific patterns of neuroimaging abnormalities (Marquand et al., 2016; Wolfers & Beckmann, 2020; Wolfers et al., 2018; Zabihi et al., 2019). Normative modeling characterizes individual-level differential patterns of MRI metrics by assessing extreme deviations from normal expectations constructed based on demographic information from healthy cohorts (Marquand et al., 2016; Wolfers et al., 2018). This approach has demonstrated effectiveness in revealing personalized patterns of gray matter morphological abnormalities across a range of psychiatric disorders, including schizophrenia, MDD, autism, and attention deficit hyperactivity disorder (Marquand et al., 2016; Wolfers & Beckmann, 2020; Wolfers et al., 2018; Zabihi et al., 2019). In the context of MDD, researchers have identified individualized gray matter morphological abnormalities that define two reproducible biotypes, showing divergent associations with responses to antidepressant treatments (Li et al., 2023; Shao et al., 2023). These studies contribute to our understanding of the heterogeneity and offer insights for precision medicine within MDD. Moving beyond structural brain abnormalities, MDD individuals exhibit alterations in spontaneous neural activity, typically measured through the amplitude of low-frequency fluctuations (ALFF) (Zang et al., 2007). Abnormal ALFF has been reported in depressed and remitted patients, as well as subthreshold individuals, signifying a trait-related marker of vulnerability to MDD (Huang et al., 2021; Jing et al., 2013) and demonstrating modulation by treatment (Huang et al., 2021; Kong et al., 2017; Wall et al., 2023; Xiao et al., 2024). However, the heterogeneity of spontaneous neural activity abnormalities in MDD is understudied.

Furthermore, previous examinations of individual-level abnormalities in neuroimaging metrics face certain constraints. First, patients with psychiatric disorders exhibit extensive variations in individual-level abnormal patterns of neuroimaging metrics, to the extent that a regional extreme deviation is shared by at most 7% of cases with the same diagnosis (Wolfers & Beckmann, 2020; Wolfers et al., 2018; Zabihi et al., 2019). These studies fall to reconcile the contradiction between the notable variations and phenotypic similarities among cases with identical diagnoses (Segal et al., 2023). In a prior study, the authors addressed the heterogeneity among individualized gray matter morphological abnormalities in MDD from a dimensional perspective. Their findings suggested that these individualized abnormalities could be depicted as a unique and linear weighted sum of shared differential factors in MDD. This approach effectively reconciled the contradiction between interindividual variability in neuroimaging abnormalities and the observed phenotypic similarities (Han et al., 2023a). In the current study, we hypothesize that individualized ALFF abnormalities are also constituted by differential factors shared by MDD. Second, the transcriptional and molecular substrates underlying individualized ALFF abnormalities remain elusive. For instance, the neuroinflammation hypothesis of depression posits that an elevated inflammatory response induced by factors such as stress exerts direct neurotoxic effects on the brain, leading to structural and functional changes (Berk et al., 2013; Eisenberger et al., 2010; Han & Ham, 2021; Troubat et al., 2021). Transcriptional profiles of inflammation-related genes have shown associations with cortical thickness decrease in psychiatric disorders, such as schizophrenia (Cui et al., 2023). Additionally, neurotransmitter dysfunction contributes to cortical abnormalities in psychiatric disorders including MDD (Hansen et al., 2022). Investigating the transcriptional and molecular bias associated with individualized ALFF abnormalities helps bridge the gap between neuroimaging and biological mechanisms in MDD. Third, a notable limitation in relevant studies is the relatively small sample size, limiting the reproducibility and reliability.

**Figure 1.**
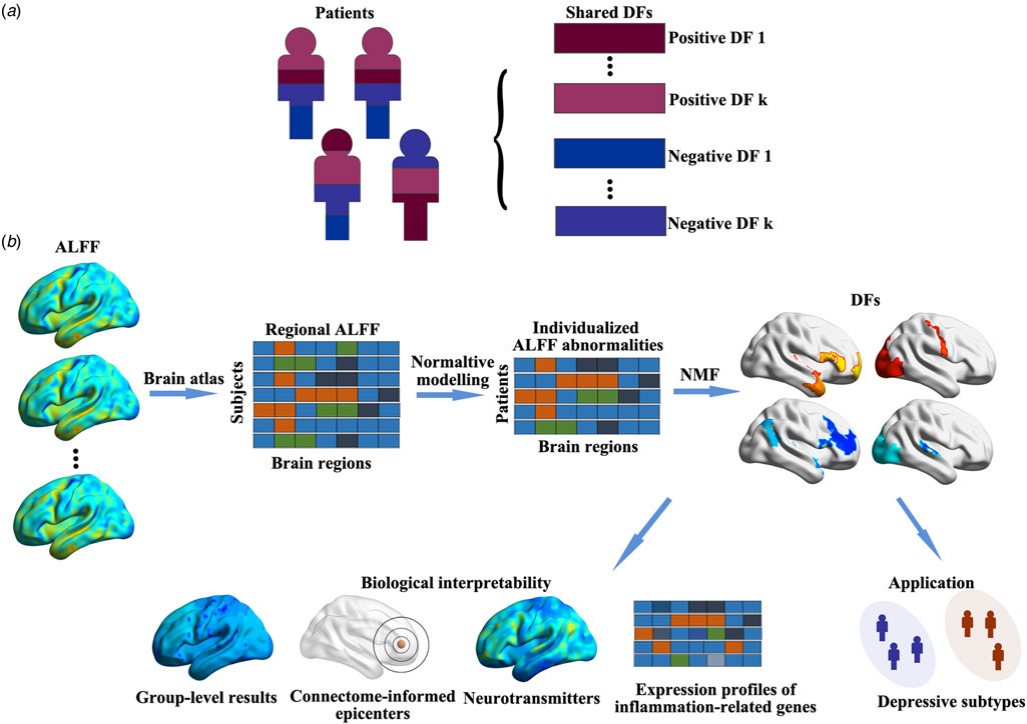
Workflow of this study. In (a), we propose that individualized ALFF abnormalities can be expressed as a linear weighted sum of shared differential factors (DFs) in MDD. Moving to (b), the regional ALFF abnormalities are derived through normative modeling and further deconstructed into DFs using NMF. To enhance the biological interpretability of these identified DFs, we explore their associations with group-level results, connectome-informed epicenters, the distribution of neurotransmitters, and expression profiles of inflammation-related genes. Additionally, we utilize factor compositions to identify potential subtypes.

To address these limitations, this study leveraged extensive data from two large multi-center datasets, comprising 2424 patients with MDD and 2183 healthy controls (HCs) to parse interindividual heterogeneity in ALFF abnormalities in MDD. Inspired by a previous research on gray matter morphological abnormalities (Han et al., 2023a), we postulated that individualized ALFF abnormalities could be expressed as linear weighted sum of shared differential factors in MDD (Fig. 1a). We employed normative modeling trained with HCs to deduce individualized ALFF abnormalities in patients and then dissected them into differential factors using non-negative matrix factorization (NMF) (Han et al., 2023a). A series of sensitivity analyses were conducted to evaluate the robustness and generalizability of these identified factors. Subsequently, we explored the relevance of these factors to both group-level outcomes and clinical characteristics. To gain deeper insights into these factors, we examined their distinct associations with the distribution of neurotransmitter receptors/transporters, transcriptional profiles of inflammation-related genes, and connectome-informed epicenters. Notably, our findings revealed that these factor compositions facilitated the identification of four reproducible subtypes, each exhibiting unique patterns of ALFF abnormalities and clinical characteristics (Fig. 1b).

## Methods

### Samples

This study included two extensive multi-center datasets comprising 2424 patients with MDD and 2183 HCs sourced from the Disease Imaging Data Archiving-Major Depressive Disorder Working Group (DIDA-MDD) and REST-meta-MDD consortium. The DIDA-MDD encompassed nine research sites (Sun et al., 2023). Following rigorous quality control for both clinical and imaging data (Sun et al., 2023), the final sample included 1148 patients with MDD (age: 33.83 ± 22.17 years, females: 58.62%) and 1079 HCs (age: 33.96 ± 19.25 years, females: 56.81%). Patients received diagnoses from experienced psychiatrists based on the Diagnostic and Statistical Manual of Mental Disorders-IV (DSM-IV) criteria for MDD, without comorbidities with other psychiatric disorders or current substance use. The severity of symptoms in patients was assessed using the Hamilton Depression Rating Scale (HAMD) (Hamilton, 1960). HCs and first-degree relatives had no current or lifetime history of psychiatric disorders, and all participants had no nervous system diseases, cardiovascular diseases, or a history of trauma or brain surgery. Further details are thoroughly described elsewhere (Sun et al., 2023; Xia et al., 2022).

The validation dataset was sourced from the REST-meta-MDD consortium (http://rfmri.org/REST-meta-MDD) (Chen et al., 2022; Yan et al., 2019), comprising 25 research sites with 1276 patients diagnosed with MDD (age: 36.23 ± 21.378 years, females: 63.71%) and 1104 HCs (age: 36.15 ± 24.552 years, females: 58.06%). Patients were diagnosed by experienced psychiatrists based on DSM-IV criteria for MDD. Additional details can be found in previous studies (Chen et al., 2022; Yan et al., 2019).

All analyses were initially conducted in the discovery dataset and subsequently replicated in the validation dataset unless specified otherwise. Approval for this study was obtained from the local Research Ethics Committees, and informed written consent was obtained from all participants before the experiment. For further information on data acquisition and preprocessing, refer to the online Supplementary methods.

### Modeling individualized ALFF abnormalities

In the discovery dataset, ALFF maps were calculated with the Data Processing Assistant for Resting-State fMRI (DPARSF) protocol (version 5.4) (Chao-Gan & Yu-Feng, 2010). In the validation dataset, we utilized the released ALFF maps from the REST-meta-MDD consortium (http://rfmri.org/REST-meta-MDD). Mean ALFF values were calculated for each brain region defined in the brain connectome atlas (Fan et al., 2016). Combat harmonization was independently performed in each dataset to minimize site effect on ALFF values (Johnson, Li, & Rabinovic, 2007) in each dataset independently. Combat, a technique capable of removing unwanted site variation while preserving biological variability, has been widely employed in neuroimaging studies to mitigate site effects on imaging metrics (Fortin et al., 2017, 2018; Shao et al., 2023).

As done in previous studies (Han et al., 2023a; Marquand et al., 2016; Wolfers et al., 2018), individualized ALFF abnormalities were calculated. Specifically, for each brain region, a Gaussian process regression was trained to estimate the normative range of ALFF values based on age and sex in HCs. The trained model was then applied to each patient, generating a *Z*-score (individualized ALFF abnormality). The *Z*-score represents the degree of deviation from the healthy reference population's normative range, where a positive *Z*-score indicates a higher ALFF value than HCs in patients with MDD and vice versa. While normative modeling has shown robust predictive capabilities regarding gray matter morphology, its effectiveness in predicting ALFF values remains unexplored. Therefore, the performance of normative modeling on ALFF was thoroughly evaluated using the following strategies: (1) 10-fold cross-validation (repeat 100 times); (2) leave-one-site-out cross-validation, where one set served as the testing site and the remaining as the training set; (3) training a Gaussian process regression model based on HCs in the discovery dataset and applying it to HCs in the validation dataset, and vice versa; and (4) utilizing another brain atlas with different resolutions (automated anatomical labeling, AAL) containing 90 cortical and subcortical regions (excluding the cerebellum) (Tzourio-Mazoyer et al., 2002). Model performance was assessed by calculating the standardized mean squared error (MSE) between the true ALFF values and predicted ones (Marquand et al., 2016).

After confirming the performance of normative modeling performance, *Z*-scores were calculated for each patient using the trained model with HCs, resulting in a *Z*-score matrix for patients (number of patients × brain regions, *N* × 246).

### Modeling differential factors

First, we replicated previous findings about heterogeneity in locations of individualized neuroimaging abnormalities. We quantified regional heterogeneity in ALFF deviations as the proportion of patients showing an extreme deviation in each brain region, where regional extreme deviations were defined as |*Z*| > 2.6 (corresponding to *p* < 0.005) (Segal et al., 2023; Wolfers et al., 2018).

Given the little overlap in locations of regional individualized ALFF abnormalities (see Results), we next parsed individualized ALFF abnormalities from the perspective of dimension. We hypothesized that individualized ALFF abnormalities could be expressed as a linear combination of differential factors shared by all patients. Individualized ALFF abnormalities (*Z*-score matrix) were parsed into latent differential factors using NMF (Han et al., 2023a) defined as follows:1
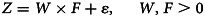
In the above equation, *Z* represents the *Z*-score matrix (with dimensions: number of patients × brain regions), *F* denotes the latent differential factors (with dimensions: number of factors (*K*) × brain regions), *W* (with dimensions: number of patients × *K*) signifies factor compositions (weights), and 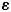 denotes the residuals. Notably, the optimal number of factors (*K*) remains unknown.

To determine the optimal number of factors (*K*) within the range of 2–10, we employed the generalizability error (GE) metric (Chen et al., 2020). The GE was defined as the mean of absolute differences between the reconstructed out-of-sample *Z*-scores, obtained using the trained NMF, and the true *Z*-scores. A lower GE indicated superior generalizability. Specifically, we randomly split patients into two halves (half 1 and 2). NMF was trained using one half (half 1) and then applied to the remaining half (half 2). The mean absolute reconstruction error (between the reconstructed and true *Z*-scores) for half 1 (*e*_11_) and half 2 (*e*_12_) were calculated. Similarly, *e*_11_ and *e*_12_ were also obtained. The GE was calculated using the following formula:2
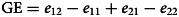
This procedure was repeated 100 times, and the optimal number was identified as the one exhibiting the lowest GE.

To evaluate the generalizability of the identified differential factors, we computed spatial correlations between factors obtained from distinct datasets. The correspondence between factors was established using the Hungarian matching algorithm (Lee et al., 2022; Zhang et al., 2016). Significance was set at *p* < 0.05, with false-discovery rate (FDR) correction.

We then assessed the extent to which the identified differential factors accounted for the variance in individualized ALFF abnormalities, and whether they explained a greater proportion of variance than chance. Specifically, for each patient, a linear regression model was constructed to quantify the extent to which differential factors explained the variance in the individualized ALFF abnormalities. To rigorously evaluate whether the identified differential factors accounted for a more substantial variance in individualized ALFF abnormalities than expected by chance, we conducted a permutation test (10 000 times). For each run individualized ALFF abnormalities were shuffled. NMF was constructed based on shuffled data to obtain differential factors, and then variance explained was determined.

### Relationship between the differential factors and group-level abnormalities

Subsequently, we explored whether the differential factors could account for the observed group-level abnormalities. Group-level differences between patients and HCs were derived through a two-sided two-sample *t* test while controlling for age, sex, site, and mean frame-wise displacement (FD) (Han et al., 2018, 2020) for each brain region. Following this, we established a multilinear model linking group-level differences (an unthresholded *t* statistic vector) and the differential factors. The significance of the model was determined through permutation testing (10 000 permutations).

To further evaluate generalizability of the relation, we constructed a multilinear model between group-level abnormalities of the validation dataset and the differential factors acquired from the discovery dataset. Likewise, we performed the same analysis in the reverse direction, establishing a multilinear model between group-level abnormalities of the discovery dataset and the differential factors obtained from the validation dataset.

### Associations between the factor compositions and clinical features

In this phase, we explored the relationships between identified differential factors and various clinical manifestations, encompassing medication status (treated or untreated), episodicity (first or recurrent), symptom severity, and illness duration. For medication status and episodicity, we assessed their impact on spatial factor profiles through spatial correlations between untreated/first-episode patients and treated/recurrent patients. Significance was set at *p* < 0.05 with FDR correction. Additionally, we examined their influence on factor compositions by comparing compositions of untreated/first-episode patients with those of treated/recurrent patients using a two-sided two-sample *t* test, controlling for age, sex, site and mean FD. Regarding symptom severity and illness duration, we computed Pearson's correlation coefficients between factor compositions and the total scores of HAMD, illness duration, and age of onset, with significance was set at *p* < 0.05 and FDR correction. To account for sex differences in depression phenotypes, we investigated differences in factor compositions between female and male patients, employing a two-sided two-sample *t* test while controlling for age, site, and mean FD. Additionally, we calculated Pearson's correlation coefficients between factor compositions and mean FD to assess the potential effects of head motion on our results.

### Association between differential factors and normal brain network

Neuroimaging studies have highlighted the impact of psychiatric disorders on brain networks. Pathological progression are also constrained by normal brain network (Shafiei et al., 2020; Wannan et al., 2019). In our investigation, we explored whether the identified differential factors were also constrained by the normal brain network, as assessed through structural covariance (SC) network. SC network characterizes the coordination of regional volumes among brain regions, potentially mirroring common developmental trajectories (Alexander-Bloch, Giedd, & Bullmore, 2013; Dong et al., 2020, 2023b; Han et al., 2023c; Yun, Jang, Kim, Jung, & Kwon, 2015), implicated in the pathology of depression (Han et al., 2023b; Kaiser, Andrews-Hanna, Wager, & Pizzagalli, 2015; Lima-Ojeda, Rupprecht, & Baghai, 2018; Sotiras et al., 2017; Yun et al., 2020).

To construct the normal SC network, we utilized another large single-center dataset (SALD), comprising 492 healthy individuals aged 19–80. Voxel-based morphometry was calculated following the recommended pipeline of the CAT 12 toolbox (http://dbm.neuro.uni-jena.de/cat12/), with additional details available elsewhere (Ashburner, 2009; Han et al., 2021a, 2021b). Then, SC network was obtained by calculating Pearson's correlation coefficients between average gray matter values of 246 brain regions controlling age, age^2^, and sex. Negative correlations were set to zero (Li et al., 2023).

We quantified the relationship between the SC network and the identified differential factors, aligning with methodologies established in prior studies (Li et al., 2023; Shafiei et al., 2020). For each differential factor (*F*), the normalized collective abnormalities/differences of structural neighbors of region *i* (*D_i_*) are obtained as follows:
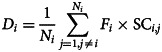
where *D_i_* represents the normalized collective abnormalities/differences of the structural neighbors of region *i*, *F*_*i*_ is the average abnormal value of region *i*, *N*_*i*_ is the number of neighbors of region *i* with an SC connection, and SC_*i*,*j*_ is the strength of SC between regions *i* and *j*. For each factor, the *D_i_* was predicted using the values of the neighboring regions. The Pearson's correlation coefficient between true values and predicted ones across all brain regions was calculated.

Furthermore, we identified putative epicenters for each differential factor. A brain region was considered as the epicenter if, along with its connected neighbors, it exhibited high abnormal values (Li et al., 2023; Shafiei et al., 2020). Brain regions were ranked based on their regional values, and SC-informed values in the ascending order. Then, the average ranking values were considered as the epicenter-likelihood rankings, and significance was determined through permutation testing (10 000 times).

### Contribution of neurotransmitter receptors/transporters to differential factors

The involvement of neurotransmitter receptor profiles in the pathology of MDD has long been established (Hansen & Shafiei, 2022). Consequently, we investigated the associations between neurotransmitter receptors/transporters profiles and the identified differential factors. To achieve this, we constructed a multilinear model between neurotransmitter receptors/transporters profiles and each differential factor. The significance of these multilinear models was evaluated through permutation testing (10 000 times), with FDR correction. The neurotransmitter receptors/transporters profiles were sourced from an atlas derived from positron emission tomography (PET) scans of 1238 healthy individuals (Hansen & Shafiei, 2022). This atlas encompasses a total of 19 unique neurotransmitter receptors, receptor-binding sites, and transporters across nine different neurotransmitter systems. The atlas includes receptors for serotonin (Hansen & Shafiei, 2022) (5HT_1A_ (Savli et al., 2012), 5HT_1B_ (Baldassarri et al., 2018; Gallezot et al., 2010; Matuskey et al., 2014; Murrough et al., 2011a, 2011b; Saricicek et al., 2015; Savli et al., 2012), 5HT_2A_ (Beliveau & Ganz, 2017), 5HT_4_ (Beliveau & Ganz, 2017), 5HT_6_ (Radhakrishnan et al., 2018, 2020), 5HTT (Beliveau & Ganz, 2017)), norepinephrine (*α*_4_*β*_2_ (Baldassarri et al., 2018; Hillmer et al., 2016), M_1_ (Naganawa et al., 2021), VAChT (Aghourian, Legault-Denis, Soucy, & Rosa-Neto, 2017; Bedard et al., 2019)), cannabinoid (CB_1_ (D'Souza et al., 2016; Neumeister et al., 2012; Normandin et al., 2015; Ranganathan et al., 2016), NET (Belfort-DeAguiar et al., 2018; Ding et al., 2010; Li et al., 2014; Sanchez-Rangel et al., 2020)), dopamine (D_1_ (Kaller et al., 2017), D_2_ (Sandiego et al., 2015; Slifstein et al., 2015; Smith et al., 2019; Zakiniaeiz et al., 2019), DAT (Dukart et al., 2018)), GABA (GABA_a_ (Nørgaard et al., 2021)), histamine (H_3_ (Gallezot et al., 2017)), glutamate (mGluR_5_ (DuBois et al., 2016; Smart et al., 2019), NMDA (Galovic et al., 2021; McGinnity et al., 2014)), and opioid (MOR (Kantonen et al., 2020)). PET images were averaged across participants within each study, registered to the MNI-ICBM 152 non-linear 2009 template, and then parcellated into 246 brain regions as defined in the brain connectome atlas (Fan et al., 2016). The average regional neurotransmitter receptor/transporter densities were *Z*-scored (Hansen & Shafiei, 2022).

To ascertain the relative importance of predictors (neurotransmitter receptors/transporters), a dominance analysis was conducted. This analysis estimates the relative importance of predictors by fitting the same multilinear model on all possible combinations of predictors (Budescu, 1993). Each predictor is assigned a total dominance value, representing its relative importance in the model (Azen & Budescu, 2003; Budescu, 1993; Hansen & Shafiei, 2022). Additionally, we categorized receptors into excitatory and inhibitory receptors and determined their cumulative contributions to differential factors by summing the total dominance values, respectively.

### Association between differential factors and transcriptional profiles of inflammation-related genes

We also explored potential associations between the identified differential factors and gene expression patterns linked to inflammation. Fourteen inflammation-related genes identified in a previous review (Keller et al., 2014) were chosen, and their transcriptional data were sourced from the Allen Human Brain Atlas (AHBA) (http://human.brainmap.org/) (Hawrylycz et al., 2012). The raw expression data for inflammation-related genes underwent processing using the AHBA-recommended pipeline (Dong et al., 2023a; Hawrylycz et al., 2012). For each gene, expression data were averaged across the six donors and normalized to *Z*-scores across 246 brain regions. Subsequently, the average expression data for inflammation-related genes were compiled into a 246 × 1 expression data vector.

Following this, we calculated the Pearson's correlation coefficient between the spatial patterns of the identified differential factors and the transcriptional profiles of inflammation-related genes. Significance was assessed through permutation testing (10 000 times) and corrected using FDR correction.

### Subtyping patients based on factor compositions

Next, we investigated whether factor compositions helped to uncover potential subtypes. Employing a Gaussian mixture model (GMM), we utilized factor compositions as features to identify these subtypes. The optimal number of subtypes was determined using Bayesian information criterion (BIC) within the range of 2–10.

To assess the stability and replicability of the subtyping results, we applied a trained GMM using the discovery dataset to the validation dataset, and vice versa. The adjusted Rand index (ARI) between predicted subtype labels and true ones was calculated.

Then, we characterized the subtypes using clinical features and ALFF abnormalities. Specifically, regional ALFF differences for each subtype compared to HCs were examined using a two-tailed two-sample *t* test, controlling for age, sex, site, and mean FD. Statistical significance was set at *p* < 0.05 with Bonferroni correction for multiple comparisons. This rigorous correction method was applied due to subtypes demonstrating ALFF differences almost spanning the whole brain.

Additionally, we investigated clinical differences between subtypes, including symptom severity and illness duration. Other clinical characteristics were not considered due to their unavailability for most patients in certain subtype. Statistical significance was set at *p* < 0.05 with FDR correction for multiple comparisons.

## Results

### Demographics and clinical characteristics

Demographics and clinical characteristics are presented in online Supplementary Table S1.

### The robustness and generalizability of Gaussian process regression in predicting ALFF

We first assessed the performance of Gaussian process regression model with a series of strategies. (1) Results from 10-fold cross-validation highlighted the ability of Gaussian process regression in predicting ALFF values for unseen individuals. Online Supplementary Fig. S1 displays the spatial distribution of average MSE values across 100 runs of 10-fold cross-validation, reflecting the consistency between true and predicted ALFF values. (2) The spatial distribution of MSE values obtained from leave-one-site-out cross-validation is illustrated in online Supplementary Fig. S2. The relatively small MSE values affirm the generalizability of Gaussian process regression for independent sites. (3) Additionally, a Gaussian process regression was trained based on HCs in the discovery dataset and then used to infer ALFF values for HCs in the validation dataset, and vice versa. The spatial distributions of MSE values between predicted ALFF values and true ones of the discovery and validation datasets are shown in online Supplementary Fig. S3. These results affirmed the generalizability of Gaussian process regression again. (4) Furthermore, we validated the aforementioned results using the AAL atlas and the findings remained largely unchanged, as depicted in online Supplementary Figs. S1–S3. These outcomes confirm the robustness and generalizability of Gaussian process regression in predicting ALFF.

### Four differential factors underlying individualized ALFF abnormalities in MDD are identified

In line with previous studies, patients with MDD exhibited considerable heterogeneity, with the maximum overlap percentage not surpassing 9% in the discovery dataset (6% in the validation dataset, online Supplementary Fig. S4).

**Figure 2.**
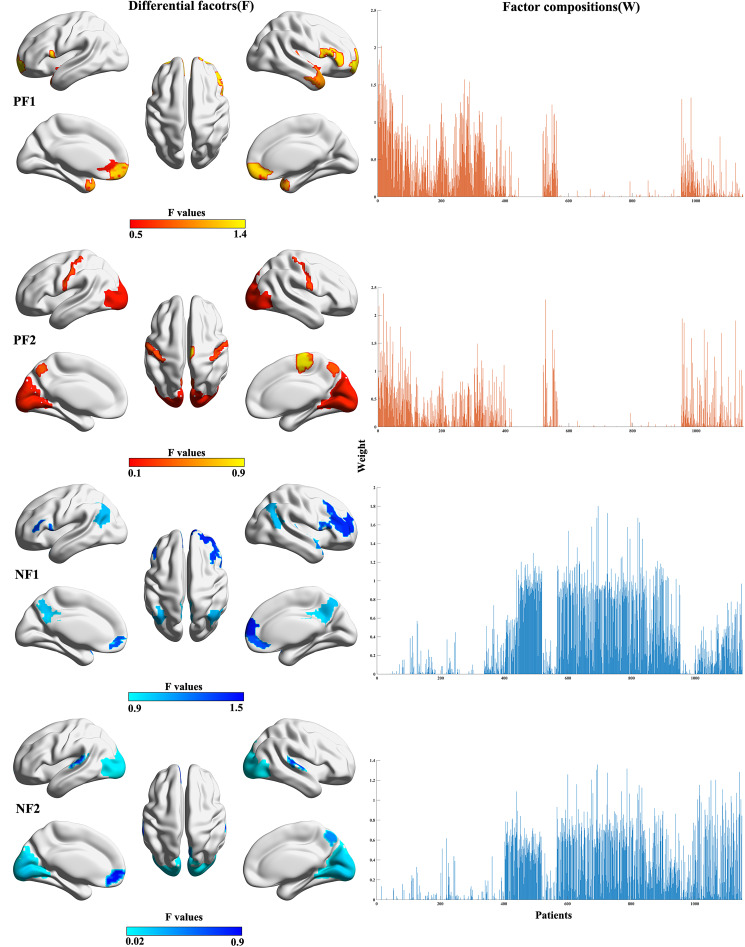
Most representative regions (the top 10% of 246 brain regions according to *F* values) of the identified differential factors and the corresponding factor composition (W) of patients. PF1, positive factor 1; PF2, positive factor 2; NF1, negative factor 1; NF2, negative factor 2.

Subsequently, we deconstructed individualized ALFF abnormalities into latent differential factors using NMF. When *K* = 2, the GE reached a minimum for both positive and negative factors (online Supplementary Fig. S5). This result suggests the existence of two positive and two negative differential factors underlying ALFF abnormalities. The most representative regions (the top 10% of 246 brain regions based on *F* values) of the identified factors and the factor compositions of patients are presented in Fig. 2. Positive factor 1 primarily involved the ventromedial prefrontal cortex/anterior cingulate cortex and superior temporal gyrus. Positive factor 2 predominantly encompassed the visual cortex, precuneus, precentral gyrus, and medial frontal gyrus. Negative factor 1 covered brain regions mainly situated in the default mode network, including the middle frontal gyrus, medial frontal gyrus, superior temporal gyrus, precuneus, angular gyrus, and insula. Negative factor 2 included the superior temporal gyrus, visual cortex, and medial frontal gyrus. The identified differential factors and the factor compositions of patients in the validation dataset are displayed in online Supplementary Fig. S6.

The identified differential factors using different datasets exhibited significant spatial correlations, with Pearson's correlation coefficients of 77.28 × 10^−2^ (95% confidence interval [CI] 71.70 × 10^−2^ to 81.87 × 10^−2^), 84.98 × 10^−2^ (95% CI 81.09 × 10^−2^ to 88.12 × 10^−2^), 66.66 × 10^−2^ (95% CI 59.08 × 10^−2^ to 73.08 × 10^−2^), 80.38 × 10^−2^ (95% CI 75.46 × 10^−2^ to 84.40 × 10^−2^) for positive factor 1, positive factor 2, negative factor 1, and negative factor 2, respectively (online Supplementary Fig. S7). All FDR-corrected *p* < 1.00 × 10^−4^.

On average, these differential factors accounted for 57.19% (±6.84%, adjusted *R*^2^) of the variation in the individualized ALFF abnormalities. The results of the permutation test indicated that these factors accounted for a significantly greater proportion of variance than would be expected by chance, with a permutation *p* < 1.00 × 10^−4^. This outcome suggests that the individualized ALFF abnormalities were substantially captured by these factors.

### Relationship between the differential factors and group-level abnormalities

At the group level, patients exhibited a widespread decrease in ALFF across almost the entire brain (FDR-corrected *p* < 0.05). The group-level ALFF abnormalities of the discovery and validation datasets are presented in online Supplementary Fig. S8. Subsequently, we explored the relationship between the identified differential factors and group-level abnormalities (an unthresholded *t* statistic vector) by constructing a multilinear model. The goodness-of-fit (adjusted *R*^2^) of the model was 0.71 (*F*-statistic = 147, permutation *p* < 1.00 × 10^−4^). This result was replicated in the validation dataset, with a goodness-of-fit (adjusted *R*^2^) of 0.68 (*F*-statistic = 133, permutation *p* < 1.00 × 10^−4^).

Additionally, the identified differential factors in the discovery dataset demonstrated a significant association with group-level abnormalities in the validation dataset, yielding a goodness-of-fit of 0.52 (*F*-statistic = 67.30, permutation *p* < 1.00 × 10^−4^). Conversely, when evaluated in the reverse direction, the goodness-of-fit was 0.46 (*F*-statistic = 53.20, permutation *p* < 1.00 × 10^−4^).

### Associations between differential factors and clinical features

**Figure 3.**
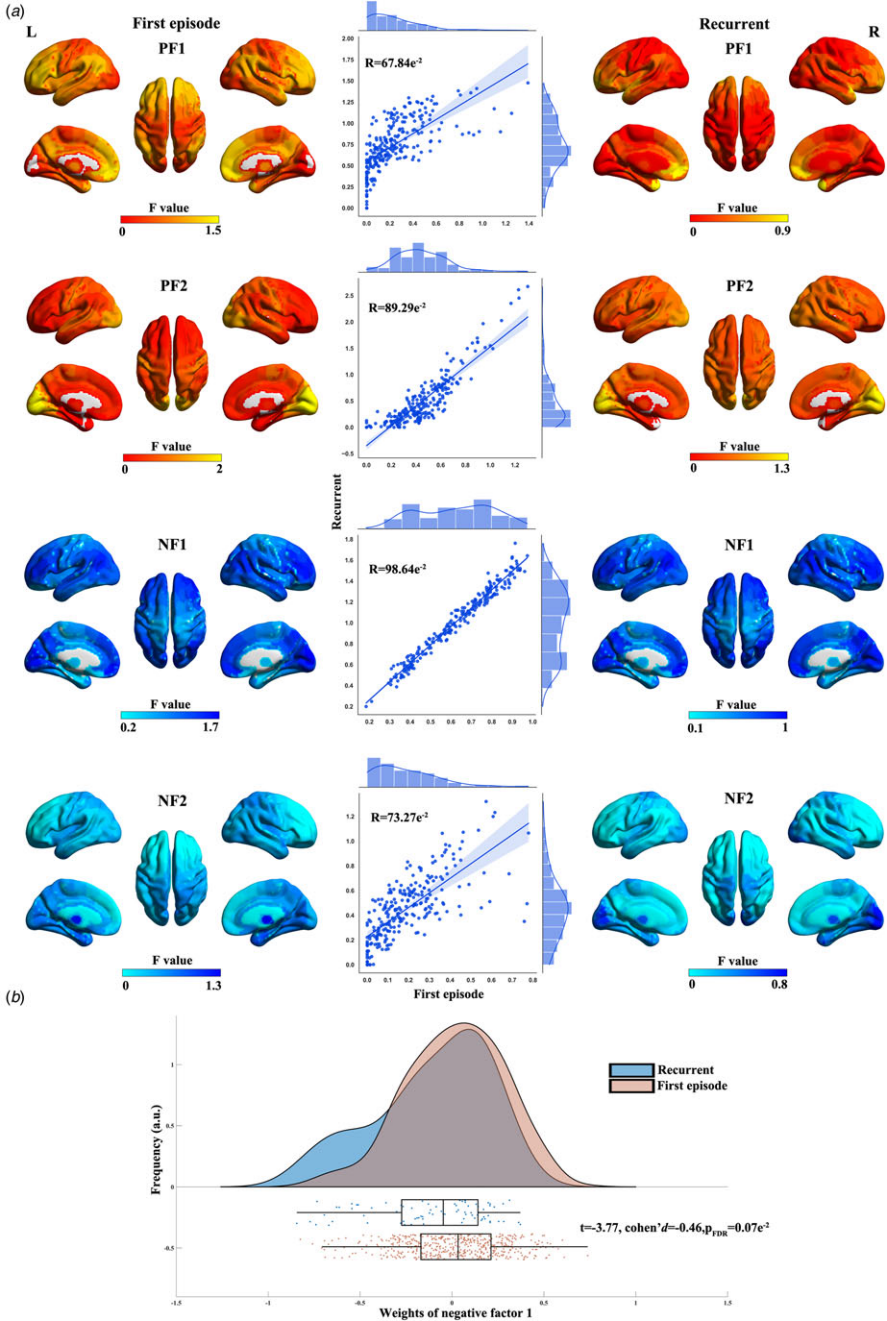
Impact of episodicity on the identified differential factors. (a) Spatial correlations between the identified differential factors using first-episode patients and those using recurrent patients. All FDR-corrected *p* < 1.00 × 10^−4^. (b) Factor composition differences between recurrent and first-episode patients. PF1, positive factor 1; PF2, positive factor 2; NF1, negative factor 1; NF2, negative factor 2.

We examined the association between the differential factors and clinical manifestations. First, we investigated the impact of episodicity (first-episode or recurrent) on profiles of differential factors. The spatial correlations between identified differential factors using first-episode patients and those using recurrent patients were 67.48 × 10^−2^ (95% CI 60.04 × 10^−2^ to 73.76 × 10^−2^), 89.29 × 10^−2^ (95% CI 86.44 × 10^−2^ to 91.57 × 10^−2^), 98.64 × 10^−2^ (95% CI 98.26^−2^ to 98.94 × 10^−2^), 73.27 × 10^−2^ (95% CI 66.90 × 10^−2^ to 78.58 × 10^−2^) for positive factor 1, positive factor 2, negative factor 1, and negative factor 2, respectively (Fig. 3a). All FDR-corrected *p* < 1.00 × 10^−4^. Regarding factor compositions, recurrent patients exhibited significantly lower weights of negative factor 1 than first-episode patients (*t* = −3.77, Cohen's *d* = −0.46, FDR-corrected *p* = 0.07 × 10^−2^, Fig. 3b). These results were replicated in the validation dataset (see online Supplementary results and Fig. S9).

**Figure 4.**
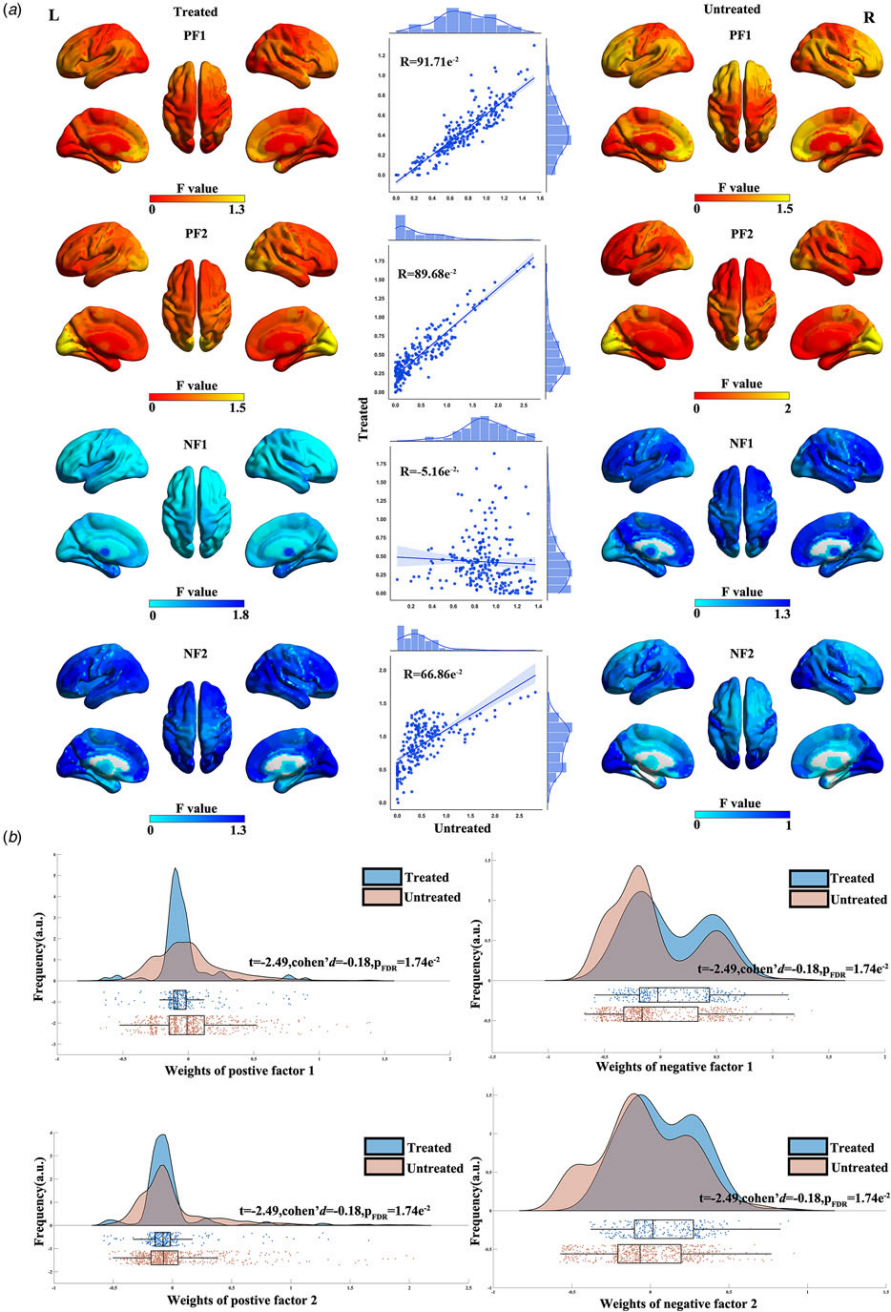
Impact of medication on the identified differential factors. (a) Spatial correlations between the identified differential factors using first-episode patients and those using recurrent patients. All FDR-corrected *p* < 1.00 × 10^−4^. (b) Factor composition differences between recurrent and first-episode patients. PF1, positive factor 1; PF2, positive factor 2; NF1, negative factor 1; NF2, negative factor 2.

Next, we explored the impact of medication (treated or untreated) on the identified differential factors. Spatial correlations between identified differential factors using untreated patients and those using treated patients were 91.71 × 10^−2^ (95% CI 89.47 × 10^−2^ to 93.49 × 10^−2^, FDR-corrected *p* < 1.00 × 10^−4^), 89.68 × 10^−2^ (95% CI 86.92 × 10^−2^ to 91.88 × 10^−2^, FDR-corrected *p* < 1.00 × 10^−4^), −5.16 × 10^−2^ (95% CI −17.55 × 10^−2^ to 7.40 × 10^−2^, uncorrected *p* = 42.07 × 10^−2^), 66.86 × 10^−2^ (95% CI 59.31 × 10^−2^ to 73.24 × 10^−2^, FDR-corrected *p* < 1.00 × 10^−4^) for positive factor 1, positive factor 2, negative factor 1, and negative factor 2, respectively (Fig. 4a). Regarding factor compositions, treated patients exhibited significantly lower weights of positive factors (*t* = −2.49, Cohen's *d* = −0.18, FDR-corrected *p* = 1.74 × 10^−2^ for positive factor 1 and *t* = −2.12, Cohen's *d* = −0.15, FDR-corrected *p* = 3.46 × 10^−2^ for positive factor 2) and higher weights of negative factors (*t* = 5.21, Cohen's *d* = 0.38, FDR-corrected *p* < 1.00 × 10^−4^ for negative factor 1 and *t* = 5.23, Cohen's *d* = 0.38, FDR-corrected *p* < 1.00 × 10^−4^ for negative factor 2) than untreated patients (Fig. 4b). These results remained largely unchanged in the validation dataset (see online Supplementary results and Fig. S10).

There were no significant differences between female and male patients in terms of factor compositions, as indicated by the *t* statistics of −0.93 (uncorrected *p* = 0.35), −0.73 (uncorrected *p* = 0.46), 1.09 (uncorrected *p* = 0.28), and −0.04 (uncorrected *p* = 0.97) for positive factor 1, positive factor 2, negative factor 1, and negative factor 2, respectively.

The total scores of HAMD were positively correlated with factor compositions of positive factors and negatively correlated with those of negative factors, with the Pearson's correlation coefficients of 14.41 × 10^−2^ (95% CI 8.48 × 10^−2^ to 20.25 × 10^−2^, FDR-corrected *p* < 1.00 × 10^−4^), 12.21 × 10^−2^ (95% CI 6.15 × 10^−2^ to 17.99 × 10^−2^, FDR-corrected *p* = 0.04 × 10^−2^), −11.22 × 10^−2^ (95% CI −17.11^−2^ to −5.25 × 10^−2^, FDR-corrected *p* = 0.10 × 10^−2^), −9.58 × 10^−2^ (95% CI −15.50 × 10^−2^ to −3.59 × 10^−2^, FDR-corrected *p* = 0.53 × 10^−2^) for positive factor 1, positive factor 2, negative factor 1, and negative factor 2, respectively (online Supplementary Fig. S11). We did not observe any significant correlations between factor compositions and mean FD (all uncorrected *p* > 0.05), excluding the potential effects of head motion on our results.

### Differential factors are informed by normal SC network and demonstrate distinct SC-informed epicenters

**Figure 5.**
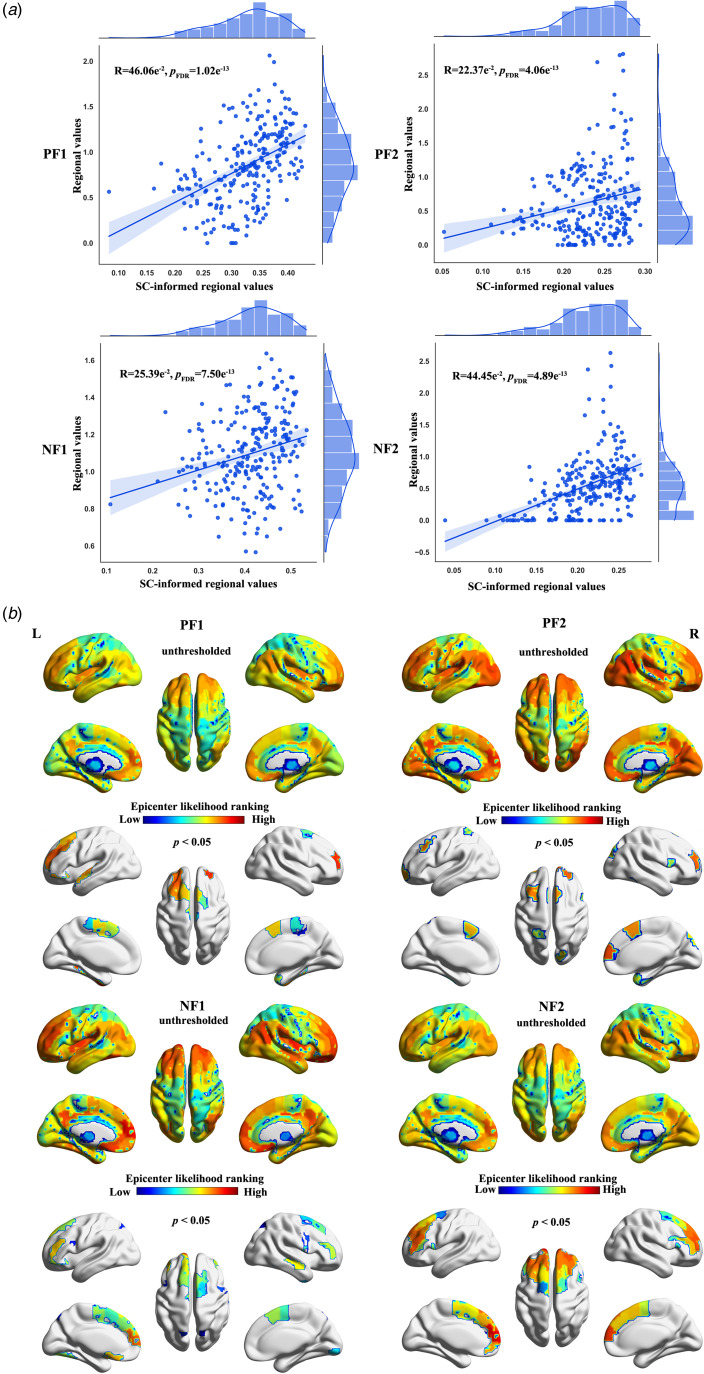
Association between the identified differential factors and normal SC network. (a) Pearson's correlation coefficients between regional values and the normalized collective abnormalities/differences of SC-informed values for each differential factor. (b) The distributions of putative epicenters are illustrated for differential factors. PF1, positive factor 1; PF2, positive factor 2; NF1, negative factor 1; NF2, negative factor 2.

The Pearson's correlation coefficients between regional values and the normalized collective abnormalities/differences of structural neighbors were 46.06 × 10^−2^ (95% CI 35.60 × 10^−2^ to 55.38 × 10^−2^, FDR-corrected *p* = 1.02 × 10^−13^), 22.37 × 10^−2^ (95% CI 10.15 × 10^−2^ to 33.93 × 10^−2^, FDR-corrected *p* = 4.06 × 10^−4^), 25.39 × 10 × 10^−2^ (95% CI 13.30 × 10^−2^ to 36.73 × 10^−2^, FDR-corrected *p* = 7.50 × 10^−5^), 44.45 × 10^−2^ (95% CI 33.83 × 10^−2^ to 53.96 × 10^−2^, FDR-corrected *p* = 4.89 × 10^−13^) for positive and negative factors, respectively. These findings indicate that differential factors are informed by normal SC network (Fig. 5a).

The average ranking values unveiled distinct epicenters for the identified differential factors. Specifically, for positive factor 1, epicenters were discerned in the superior frontal gyrus, fusiform, superior temporal gyrus, and paracentral lobule. Positive factor 2 exhibited epicenters in the superior and middle frontal gyrus, superior temporal gyrus, and inferior temporal gyrus, all marked by significantly high mean rankings. In the case of negative factor 1, epicenters were identified in the middle and inferior frontal gyrus, temporal gyrus, occipital gyrus, and precentral gyrus, each displaying significantly higher mean rankings. For negative factor 2, epicenters were concentrated in the frontal gyrus (see Fig. 5b). All permutation *p* < 0.05. Furthermore, these results were successfully replicated in the validation dataset, as detailed in the online Supplementary results and depicted in online Supplementary Fig. S12.

### Contribution of neurotransmitter receptors/transporters to differential factors

**Figure 6.**
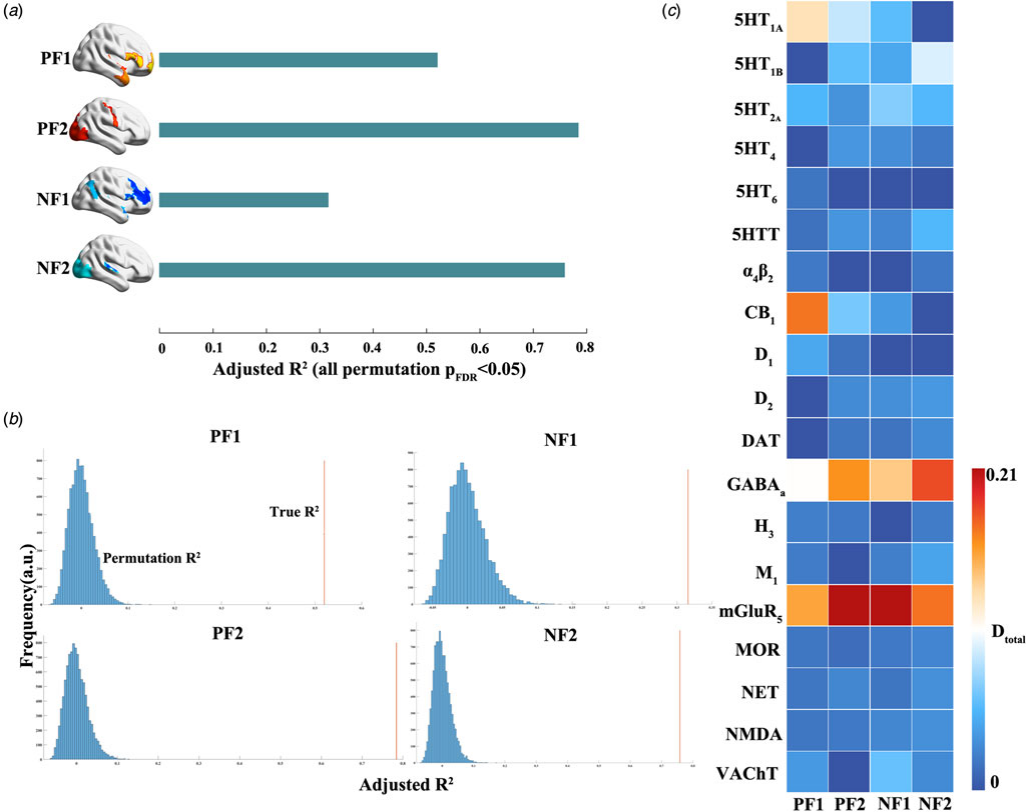
Association between neurotransmitter receptors/transporters and the identified differential factors. (a) We construct four separate multilinear models of neurotransmitter receptors/transporters and each differential factor. The corresponding model goodness-of-fit (adjusted *R*^2^) is shown in the bar plot. (b) The permutation results of multilinear models. (c) The relative importance of the predictors for each multilinear model using dominance analysis. The total dominance values, measuring the relative importance of the predictors, are shown. PF1, positive factor 1; PF2, positive factor 2; NF1, negative factor 1; NF2, negative factor 2.

Then, we investigated the association between neurotransmitter receptors/transporters and the identified differential factors by fitting four multilinear models of spatial distributions of receptors/transporters and each differential factor. The model goodness-of-fit (adjusted *R*^2^) were 0.52 (*F*-statistic (246 226) = 15.00), 0.78 (*F*-statistic (246 226) = 47.80), 0.32 (*F*-statistic (246 226) = 6.95), and 0.76 (*F*-statistic (246 226) = 41.50) for positive factor 1, positive factor 2, negative factor 1, and negative factor 2, respectively. All FDR-corrected permutation *p* < 1.00 × 10^−4^. The dominance analysis results showed that: M_1_ played an import role for all differential factors. DAT was important for factors except for positive factor 1. A_4_B_2_ was specifically important for positive factor 1 (Fig. 6). The results were replicated in the discovery dataset (online Supplementary results and Fig. S13).

### Association between differential factors and transcriptional profiles of inflammation-related genes

**Figure 7.**
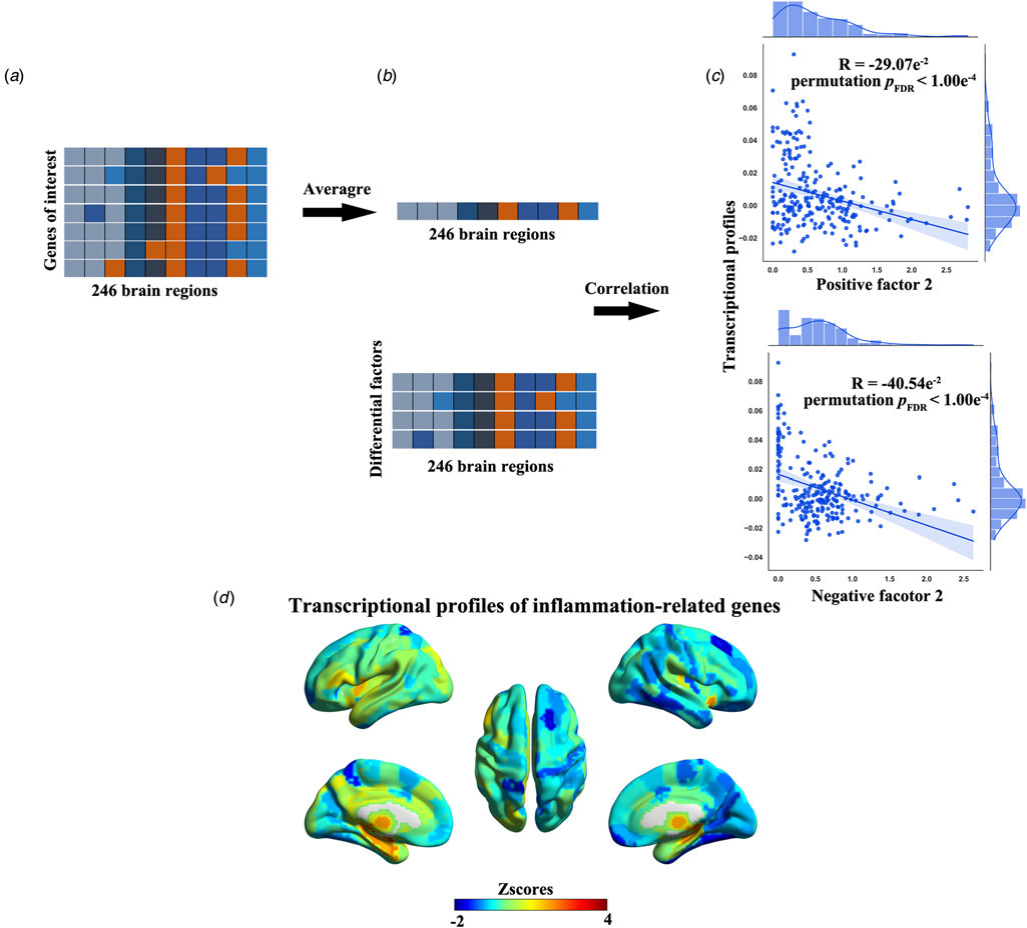
Association between differential factors and transcriptional profiles of inflammation-related genes. Regional expression profiles (*Z*-scores) of inflammation-related genes (a) are averaged (b), and then spatially correlated with patterns of the identified differential factors (c). (d) The average transcriptional profiles of inflammation-related genes are mapped to the brain.

The transcriptional profiles of inflammation-related genes were significantly correlated with patterns of positive factor 2 (*R* = −29.07 × 10^−2^, 95% CI −40.12 × 10^−2^ to −17.19 × 10^−2^, FDR-corrected permutation *p* < 1.00 × 10^−4^) and negative factor 2 (*R* = −37.96 × 10^−2^, 95% CI −48.18 × 10^−2^ to −26.73 × 10^−2^, FDR-corrected permutation *p* < 1.00 × 10^−4^) (Fig. 7). The correlations could be replicated in the validation dataset (online Supplementary results and Fig. S14).

### Factor compositions reveal four robust subtypes

**Figure 8.**
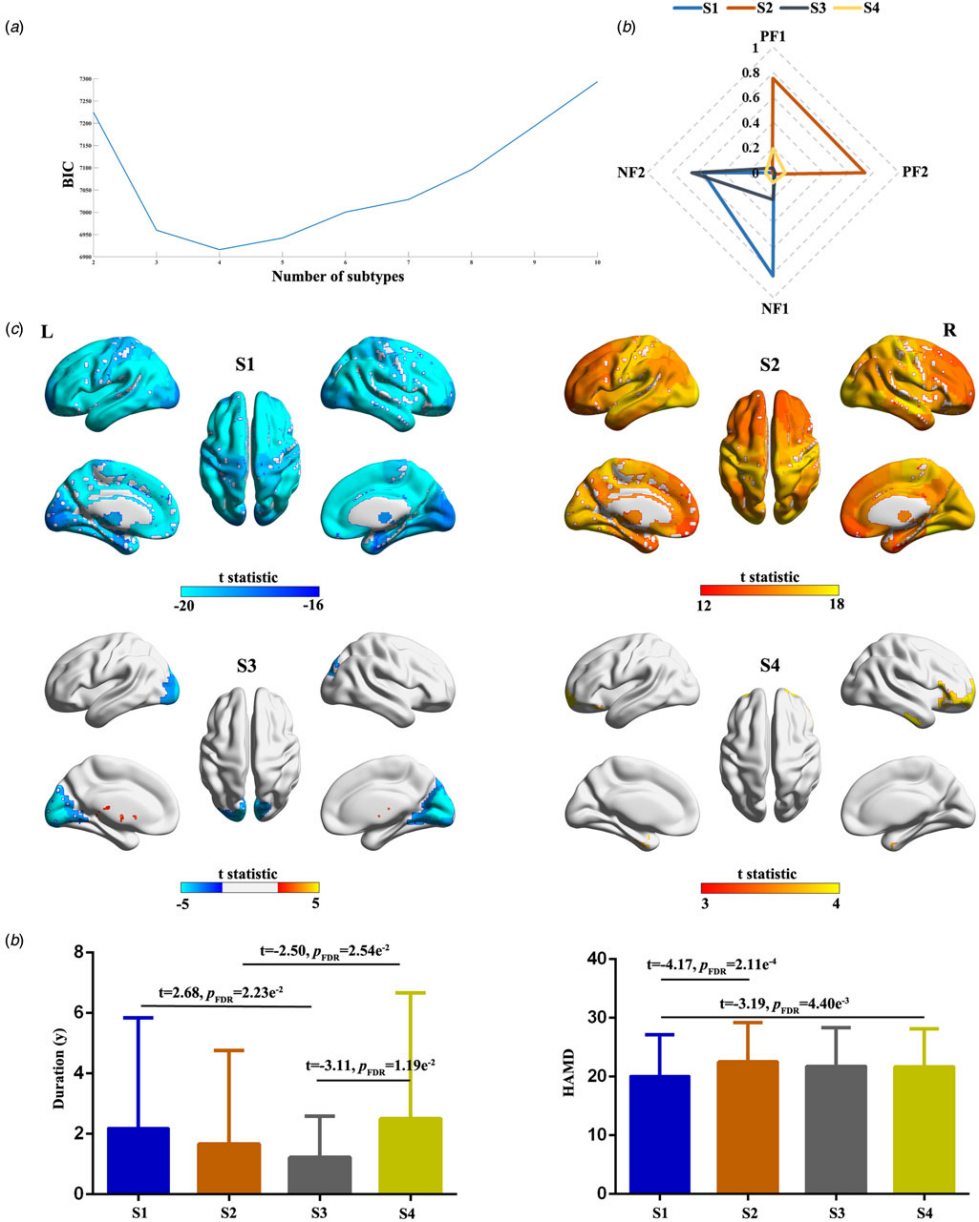
Subtyping results. (a) BIC value for each number of subtypes. (b) Average factor compositions of each subtype. (c) ALFF abnormalities of each subtype relative to healthy controls. (d) Clinical characteristic differences among subtypes. S1, subtype 1; S2, subtype 2; S3, subtype 3; PF1, positive factor 1; PF2, positive factor; NF1, negative factor 1; NF2, negative factor 2.

The factor compositions unveiled four subtypes (BIC values are presented in Fig. 8a). The average factor compositions and ALFF abnormalities for each subtype were presented in Fig. 8b. These subtypes manifested unique patterns of ALFF abnormalities relative to HCs (Fig. 8b). Specifically, subtype 1 exhibited an overall decrease in ALFF, while subtype 2 demonstrated an overall increase in ALFF compared to HCs. Subtype 3 displayed heightened ALFF in the putamen and striatum, but decreased ALFF in the calcarine, lingual, and cuneus. Subtype 4 showcased a modest increase in ALFF in the inferior temporal gyrus, parahippocampus, middle and inferior frontal gyrus. Clinical characteristic differences among subtypes are depicted in Fig. 8d. These findings were validated in the validation dataset (online Supplementary Fig. S15).

To ascertain the stability and generalizability of subtyping outcomes, a GMM trained on the discovery dataset was applied to the validation dataset, and vice versa. In the discovery dataset, the ARI between subtyping labels predicted by the GMM trained using the validation dataset and true labels was 0.52. Conversely, in the discovery dataset, the ARI between subtyping labels predicted by GMM trained using the discovery dataset and true labels was 0.39. Furthermore, ALFF abnormities between subtypes, as identified in the discovery and validation datasets, exhibited significant correlations (online Supplementary Fig. S16). These results affirm the stability and generalizability of subtyping outcomes.

## Discussion

Leveraging two extensive multi-center datasets, we identified four robust and reproducible shared factors that underlie individual-specific spontaneous neural activity abnormalities in MDD reconciling the contradiction between the notable variations in individual ALFF abnormalities and the phenotypic similarities among patients with MDD. We also elucidated how these factors were influenced by various clinical characteristics, such as medication, symptom severity, and episodicity. These factors not only explained group-level ALFF abnormalities patterns but also demonstrated unique associations with the distribution of neurotransmitter receptors/transporters, transcriptional profiles of inflammation-related genes, and connectome-informed epicenters, underscoring their neurobiological relevance. Additionally, factor compositions facilitated the identification of four distinct subtypes of depression, each characterized by unique abnormal ALFF patterns and clinical features. Notably, all these findings were successfully replicated in another dataset, thereby affirming their robustness and generalizability.

Patients with psychiatric disorders, including MDD, exhibit substantial interindividual heterogeneity in etiologies, clinical manifestations, disease courses, and treatment responses (Bondar, Caye, Chekroud, & Kieling, 2020; Drysdale et al., 2017; Krishnan & Nestler, 2008; Nguyen, Harder, Xiong, Kowalec, & Hägg, 2022). This diversity poses a significant challenge in neuroimaging studies, often leading to inconsistent findings (Han et al., 2023d; Mo et al., 2024; Zhang, Xu, Ma, Qian, & Zhu, 2024). To address this heterogeneity in neuroimaging, approaches such as the normative modeling have been proposed to infer abnormal neuroimaging metrics at the individual level (Marquand et al., 2016; Wolfers et al., 2018). Relevant neuroimaging studies have identified tremendous variability in individual-level patterns of neuroimaging metrics even among cases with the same diagnosis (Segal et al., 2023; Wang et al., 2023; Wolfers & Beckmann, 2020; Wolfers et al., 2018; Zabihi et al., 2019). Consistent with these observations, our study revealed notable heterogeneity in regional ALFF abnormalities at the individual level among patients with MDD. The interpretation of the contradiction between phenotypic similarities and heterogeneity in individualized neuroimaging metric abnormalities remains a critical question. In a recent study, Segal et al. examined the regional heterogeneity in individualized gray matter volume abnormalities among patients with psychiatric disorders. They proposed that phenotypic differences between cases are reflected in the heterogeneous localization of differential regions, while phenotypic similarities are reflected in circuits/networks comprising differential regions (Segal et al., 2023). In our study, we approached this question from a dimensional perspective, demonstrating the individualized ALFF abnormalities can be expressed as a unique and linear weighted sum of shared differential factors in MDD. In this context, shared differential factors reflect phenotypic similarities, while interindividual variability (phenotypic differences) is preserved through factor compositions. This framework effectively reconciles the contradiction between the heterogeneity in individualized ALFF abnormalities and phenotypic similarities in MDD.

The identified differential factors exhibit associations with clinical characteristics and offer an explanation for the inconsistency observed in previous group-level ALFF abnormalities in MDD. In exploring the relationship between these factors and clinical characteristics, we observed high correlations between the differential factors identified in the first-episode and recurrent patients, indicating their nature across the two subtypes. Notably, recurrent patients displayed significantly lower weights of negative factor 1 compared to first-episode patients, aligning with prior research suggesting elevated brain activity in recurrent patients, potentially as a compensatory mechanism for brain volume loss (Sun et al., 2022; Yüksel et al., 2018). We also found that weights of differential factors were significantly correlated with symptom severity. Furthermore, the identified differential factors offer an explanation for the inconsistency observed in previous group-level ALFF abnormalities in MDD (Jiao et al., 2011; Wang et al., 2012; Zhang et al., 2014). Similar to findings on individualized gray matter volume abnormalities, our study revealed both increased and decreased ALFF in most patients. However, group-level approaches only revealed decreased ALFF in patients with MDD. This underscores the potential existence of subtypes with distinct patterns of abnormal ALFF in MDD. Indeed, our study identified four reproducible subtypes with unique patterns of abnormal ALFF, shedding light on the diverse manifestations within the disorder. These results imply that the varying proportions of these subtypes within samples used in previous studies contributed to the inconsistency in findings (Jiao et al., 2011; Wang et al., 2012; Zhang et al., 2014). Additionally, we observed that group-level ALFF abnormalities could be derived from the differential factors, and this relationship remained consistent even when these factors and group-level abnormalities were based on different datasets. This establishes a connection between the identified differential factors and previous group-level findings, enhancing the interpretability of these factors.

To delve deeper into the biological mechanisms underpinning these differential factors, our study associated them with neurotransmitter receptor/transporter profiles, transcriptional profiles of genes of interest, and normal brain network. The role of neurotransmitter dysfunction in the pathology of MDD is well-established and the effectiveness of modern antidepressant drugs depends on the selective manipulation of neurotransmitter function (Hansen & Shafiei, 2022). Previous studies have highlighted the contribution of neurotransmitter receptor profiles to abnormal brain volume and spontaneous brain activity in psychiatric disorders (Hansen & Shafiei, 2022). Consistent with these findings, our results demonstrated that neurotransmitter receptor/transporter distribution account for up to 78% of the variation in differential factors. Moreover, dominance analysis revealed that they played different roles in these factors. Recent studies have proposed a connection between structural and functional brain aberrance and transcriptomes, bridging the gap between macroscale brain abnormalities and microscale architecture (Fornito, Arnatkevičiūtė, & Fulcher, 2019; Richiardi et al., 2015). In our study, we found that positive factor 2 and negative factor 2 were associated with transcriptional profiles of inflammation-related genes, shedding lights on their genetic basis. Another finding of this study was that these factors were informed by normal SC network and exhibited distinct epicenters. This aligns with the network-based spreading hypothesis, suggesting that pathological perturbations begin in focal brain regions (‘epicenters’), and then propagate to other brain regions following normal brain network architecture in psychiatric disorders (Shafiei et al., 2020; Wannan et al., 2019; Zhou, Gennatas, Kramer, Miller, & Seeley, 2012). The presence of distinct epicenters for these factors hints divergent progressions associated with disease development. Collectively, these results indicate diverse biological underpinnings and progressions for these factors.

To investigate the clinical application prospect of the identified differential factors, we employed them to uncover potential depressive subtypes. Consequently, we identified four reproducible subtypes with unique patterns of ALFF abnormalities and clinical characteristics. These subtypes revealed patterns of ALFF abnormalities that were not evident in traditional group-level approaches. Notably, subtype 2 exhibited increased ALFF spanning almost the entire brain, a feature concealed by group-level methods that typically detect decreased ALFF abnormalities. Previous studies have identified subtypes based mainly on clinical manifestations (Derks et al., 2012; Lynch et al., 2020; Mataix-Cols et al., 2004). Despite great success, clinical manifestations have a complex interplay with the underling biological substrates and are unstable with age or illness course. Identifying psychiatric subtypes from objective neuroanatomical data using data-driven approaches has gained increased popularity (Beijers et al., 2019). The current study, utilizing data-driven approaches and factor compositions as features, discovered four distinct depressive subtypes that could be consistently reproduced across two extensive multi-center datasets. This approach, rooted in objective neuroanatomical data, enhances our understanding of the biological underpinnings of depressive disorders and facilitate the development of personalized biomarkers for diagnosis and treatment (Beijers et al., 2019).

This study has some limitations. First, we did not have sufficient clinical information, such as symptom dimensions and comorbidities. The exploration of whether the identified differential factors correspond to specific symptom dimensions and are influenced by comorbidities warrants investigation in future research. Second, while significant differences in factor compositions associated with medication were observed, a quantitative analysis of the relationship between changes in factor compositions and medication variables, such as types and doses, was not conducted. Third, the inclusion of only cross-sectional data in our study restricts insights into the evolution of differential factors with disease progression. Future investigations should explore how these factors evolve over time to enhance our understanding of their dynamic nature.

This study successfully identified and characterized four robust and reproducible differential factors that underlie individual-specific spontaneous neural activity abnormalities in MDD. In this context, individualized ALFF abnormalities for each patient can be expressed as a unique linear combination of these factors. The factors not only exhibit close associations with clinical features but also reveal unique connections with the distribution of neurotransmitter receptors/transporters, transcriptional profiles of inflammation-related genes, and connectome-informed epicenters. Moreover, the use of factor compositions enables the identification of four distinct depressive subtypes, each distinguished by unique abnormal ALFF patterns and clinical features. These findings contribute new insights into the heterogeneity of spontaneous neural activity abnormalities in MDD.

## Supporting information

Han et al. supplementary material Han et al. supplementary material

## Data Availability

The discovery dataset is sourced from the Disease Imaging Data Archiving-Major Depressive Disorder Working Group (DIDA-MDD), and the discovery dataset is sourced from the REST-meta-MDD consortium (http://rfmri.org/REST-meta-MDD).

## Supplementary material

The supplementary material for this article can be found at https://doi.org/10.1017/S0033291724002617.

## Code availability

All analytical procedures in this study are based on publicly available toolkits. ALFF maps are calculated with the Data Processing Assistant for Resting-State fMRI (DPARSF) protocol (version 5.4). The normative model is freely available (GAUSSIAN PROCESS REGRESSION AND CLASSIFICATION Toolbox version 4.2, http://www.GaussianProcess.org/gpml/code). Non-negative matrix factorization is performed using the scikit-learn (version 1.3.0). Additional information is available from the corresponding author upon request.
