# Supplementary material for: Shared differential factors underlying individual spontaneous neural activity abnormalities in major depressive disorder
Source: Psychol Med. 2024 Nov 26;54(15):4316–34. doi: 10.1017/S0033291724002617 (PMC11650188; doi:10.1017/S0033291724002617)
Supplement: Han et al. supplementary material [file S0033291724002617sup001.docx]

**Supplementary results for shared differential factors underlying individual spontaneous neural activity abnormalities in major depressive disorder**

**Supplementary methods**

**Data acquisition**

Functional images were acquired using 3.0-T GE or Simens scanners with gradient-echo planar imaging sequences. The data acquisition parameters of the discovery [1, 2] and validation datasets [3, 4] were described elsewhere.

**Data preprocessing**

**Discovery dataset**

Functional MRI data were preprocessed using a standard pipeline described in previous studies. The preprocessing steps mainly included discarding the first ten time points, slice-timing and head-motion correction. Then images were normalized to standard MNI space using the EPI template, resampled to 3-mm isotropic voxels and smoothed using a 6-mm full-width at half maximum Gaussian kernel. Friston-24 head-motion parameters, white matter and cerebrospinal fluid signals were regressed out form the signals of voxels. Then, signals were filtered (0.01-0.1 Hz). Finally, a “scrubbing” procedure was performed [2, 5].

**Validation dataset**

The initial 10 time points were discarded, followed by slice-timing and head-motion correction. Then, linear trends, Friston-24 head-motion parameters, white matter and cerebrospinal fluid signals were regressed out form the signals of voxels. Then images were normalized to standard MNI space using the Diffeomorphic Anatomical Registration Through Exponentiated Lie algebra (DARTEL) tool. Finally, signals were filtered (0.01-0.1 Hz) [3, 4, 6].

**Supplementary results**

**Associations between differential factors and clinical features**

The spatial correlations between identified differential factors using first-episode patients and those using recurrent patients were 42.00e^-2^ (95% CI = [31.13e^-2^, 51.79e^-2^]), 85.61e^-2^ (95% CI = [81.87e^-2^, 88.63e^-2^]), 94.04e^-2^ (95% CI = [92.40^-2^, 95.33e^-2^]), 97.01e^-2^ (95% CI = [96.17e^-2^, 97.66e^-2^]) for positive factor 1, positive factor 2, negative factor 1, and negative factor 2, respectively (Figure S9A). All FDR-corrected *p* < 1.00e^-4^. As for the factor compositions, recurrent patients had significantly lower weights of negative factor 1 than first-episode patients (t = -2.56, cohen’s d = -0.19, FDR-corrected *p* = 4.31e^-2^, Figure S9B).

Spatial correlations between identified differential factors of untreated patients and those of treated patients were 24.27e^-2^ (95% CI = [12.13e^-2^, 35.70e^-2^], FDR-corrected p = 1.20e^-4^), 66.28e^-2^ (95% CI = [58.64e^-2^, 72.76e^-2^], FDR-corrected p < 1.00e^-4^), 84.84e^-2^ (95% CI = [80.92e^-2^, 88.01e^-2^], FDR-corrected *p* < 1.00e^-4^), 85.99e^-2^ (95% CI = [82.33e^-2^, 88.93e^-2^], FDR-corrected *p* < 1.00e^-4^) for positive factor 1, positive factor 2, negative factor 1, and negative factor 2, respectively (Figure S10A). As for the factor compositions, treated patients showed significantly higher weights of positive factors (t = 4.86, cohen’s d = 0.33, FDR-corrected p < 1.00e^-4^ for positive factor 1 and t = 5.98, cohen’s d = 0.41, FDR-corrected *p* < 1.00e^-4^ for positive factor 2) and lower weights of negative factors (t = -2.42, cohen’s d = -0.17, FDR-corrected p = 1.57e^-2^ for negative factor 1 and t = -4.35, cohen’s d = -0.30, FDR-corrected *p* < 1.00e^-4^ for negative factor 2) than untreated patients (Figure S10B).

There were no significant differences between female and male patients in terms of factor compositions, with the t statistics of -0.58 (uncorrected *p* = 0.56), -1.11 (uncorrected *p* = 0.27), -0.43 (uncorrected *p* = 67), -0.77 (uncorrected *p* = 0.44) for positive factor 1, positive factor 2, negative factor 1, and negative factor 2, respectively. We did not observe any significant correlations between factor compositions and mean FD (all uncorrected *p* > 0.05).

**Differential factors are informed by SC network and demonstrate divergent SC-informed epicenters in the validation dataset**

In the validation dataset, the Pearson’s correlation coefficients between regional values and the normalized collective deformations of structural neighbors were 24.87e^-2^ (95% CI = [12.76e^-2^, 36.25e^-2^], FDR-corrected p = 8.05e^-5^), 29.00e^-2^ (95% CI = [17.11e^-2^, 40.05e^-2^], FDR-corrected *p* = 7.52e^-6^), 28.12e^-2^(95% CI = [16.18e^-2^, 39.24e^-2^], FDR-corrected *p* = 1.00e^-5^), 38.00e^-2^ (95% CI = [26.77e^-2^, 48.22e^-2^], FDR-corrected *p* = 2.85e^-9^) for positive and negative factors, respectively. The results suggested that differential factors were informed by normal SC network (Figure S12A). Brain regions with significantly high mean ranking (potential disease epicenters) for each factor were shown in Figure S12B.

**Contribution of neurotransmitter receptors/transporters to differential factors in the discovery dataset**

The model goodness-of-fit (adjusted R^2^) were 0.67 (F-statistic (246,226) = 27.50), 0.79 (F-statistic (246,226) = 48.60), 0.49 (F-statistic (246,226) = 13.50) and 0.73 (F-statistic (246,226) = 35.20) positive factor 1, positive factor 2, negative factor 1, and negative factor 2, respectively. All FDR-corrected permutation *p* < 1.00e^-4^(Figure S13A-B). The dominance analysis results were shown in Figure S13C.

**Association between differential factors and transcriptional profiles of inflammation-related genes in the validation dataset**

In the validation dataset, the transcriptional profiles of inflammation-related genes were significantly correlated with patterns of positive factor 2 (R = -38.48e^-2^, 95% CI = [-48.64e^-2^, -27.28e^-2^], FDR corrected permutation *p* < 1.00e^-4^) and negative factor 2 (R = -40.58e^-2^, 95% CI = [-50.53e^-2^, -29.58e^-2^], FDR corrected permutation *p* < 1.00e^-4^). The results were shown in Figure S14.

**Association between differential factors and transcriptional profiles of inflammation-related genes in the validation dataset**

The transcriptional profiles of inflammation-related genes were significantly correlated with patterns of positive factor 2 (R = -38.48e^-2^, 95% CI = [-48.64e^-2^, -27.28e^-2^], FDR corrected permutation *p* < 1.00e^-4^) and negative factor 2 (R = -40.58e^-2^, 95% CI = [-50.53e^-2^, -29.58e^-2^], FDR corrected permutation *p* < 1e^-4^) (Figure S14).

**Table S1**. Demographic and clinical characteristics of participants across sites in datasets

|  | Discovery dataset | | | Validation dataset | | |
| --- | --- | --- | --- | --- | --- | --- |
|  | MDD (N=1148) | HC  (N=1079) | *p* | MDD  (N=1276) | HC  (N=1104) | *p* |
| Age, mean (SD), y | 33.83 (14.97) | 33.96 (13.87) | 0.832^a^ | 36.23 (213.78) | 36.15 (245.52) | 0.898^a^ |
| Sex (female/male) | 673/475 | 613/466 | 0.387^b^ | 813/463 | 641/462 | 0.005^b^ |
| Educational level, mean (SD), y | 10.80 (12.30) | 13.94 (11.62) |  | 11.25 (17.68) | 12.25 (24.82) | <0.001^a^ |
| Illness duration, mean (SD), y | 2.10 (3.60) | - | - | 3.04 (24.74) | - | - |
| Episode (First/Recurrent) | 512/79 | - | - | 538/282 | - | - |
| Medicated (Yes/No) | 277/622 | - | - | 408/447 | - | - |
| HAMD, mean (SD) | 21.31 (6.77) | - | - | 20.50 (58.71) | - | - |
| Age of first onset, y | 32.74 (12.37) | - | - | - | - | - |
| Mean FD, mean (SD), mm | 0.13 (0.07) | 0.12 (0.06) | 0.423^1^ | 0.14 (0.01) | 0.13 (0.01) | 0.863^a^ |

Abbreviations: MDD, Major Depressive Disorder; HC, healthy control; HAMD, Hamilton rating scale for depression; FD, framewise displacement; ^a^two-tailed two sample t test; ^b^Chi-square t-test

**Figure S1**. The performance of Gaussian process regression in inferring ALFF values in healthy controls in the discovery and validation dataset. The average standardized mean squared error (MSE) values between true ALFF values and predicted ones across 100 runs of 10–fold cross-validation are mapped on the brain. We validate the results using two different brain atlases including 246 and 90 brain regions, respectively.


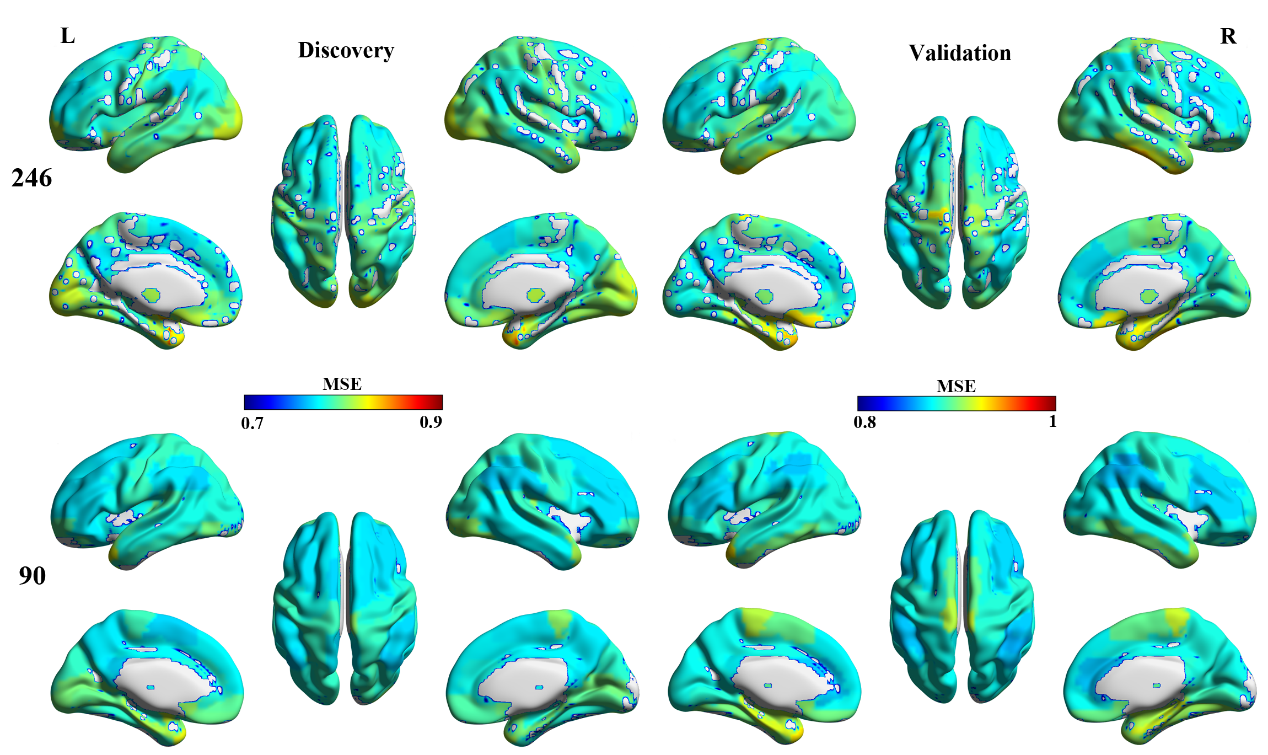


**Figure S2**. The performance of Gaussian process regression in inferring ALFF values in healthy controls in the discovery and validation dataset. The average standardized mean squared error (MSE) values between true ALFF values and predicted ones using leave-one-site-out cross-validation. We validate the results using two different brain atlases including 246 and 90 brain regions, respectively.


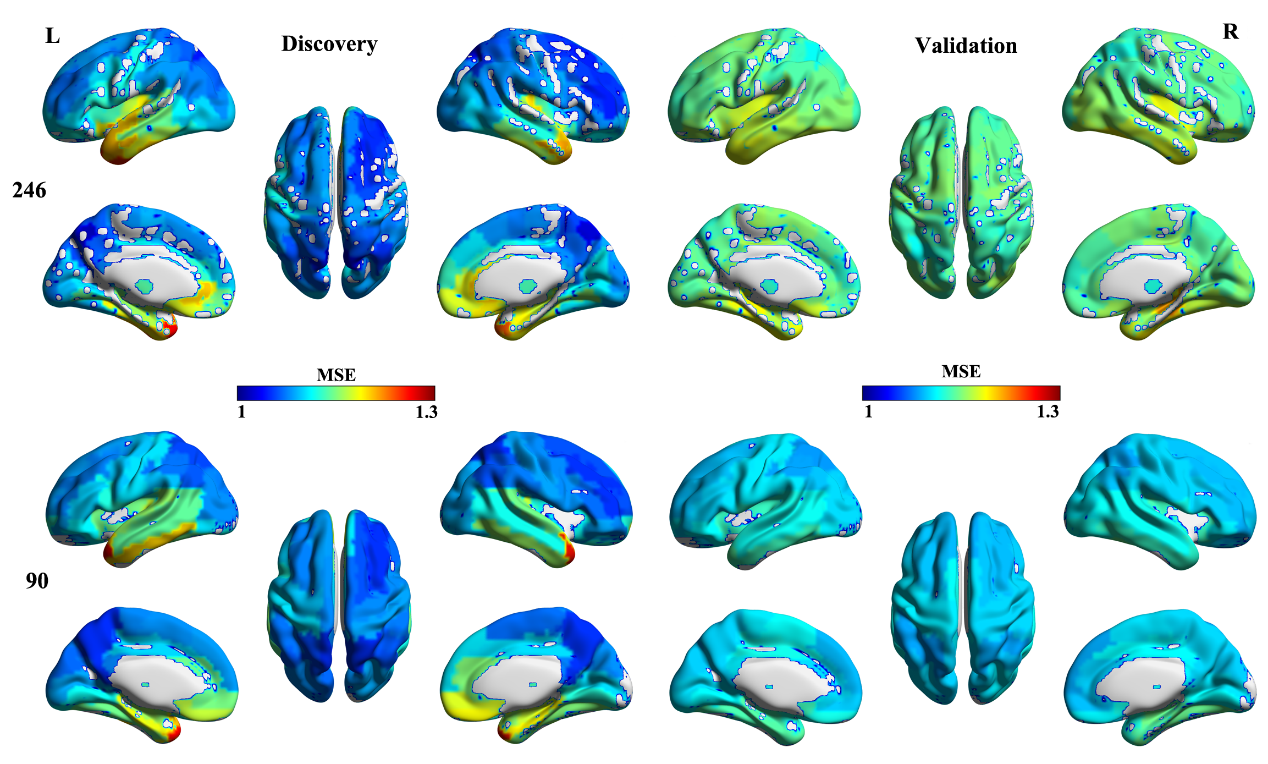


**Figure S3**. The performance of Gaussian process regression in inferring ALFF values in healthy controls in the discovery and validation dataset. The average standardized mean squared error (MSE) values between true ALFF values in the discovery dataset and predicted ones by Gaussian process regression trained using healthy controls in the validation dataset (Discovery), or the other way around (Validation). We validate the results using two different brain atlases including 246 and 90 brain regions, respectively.


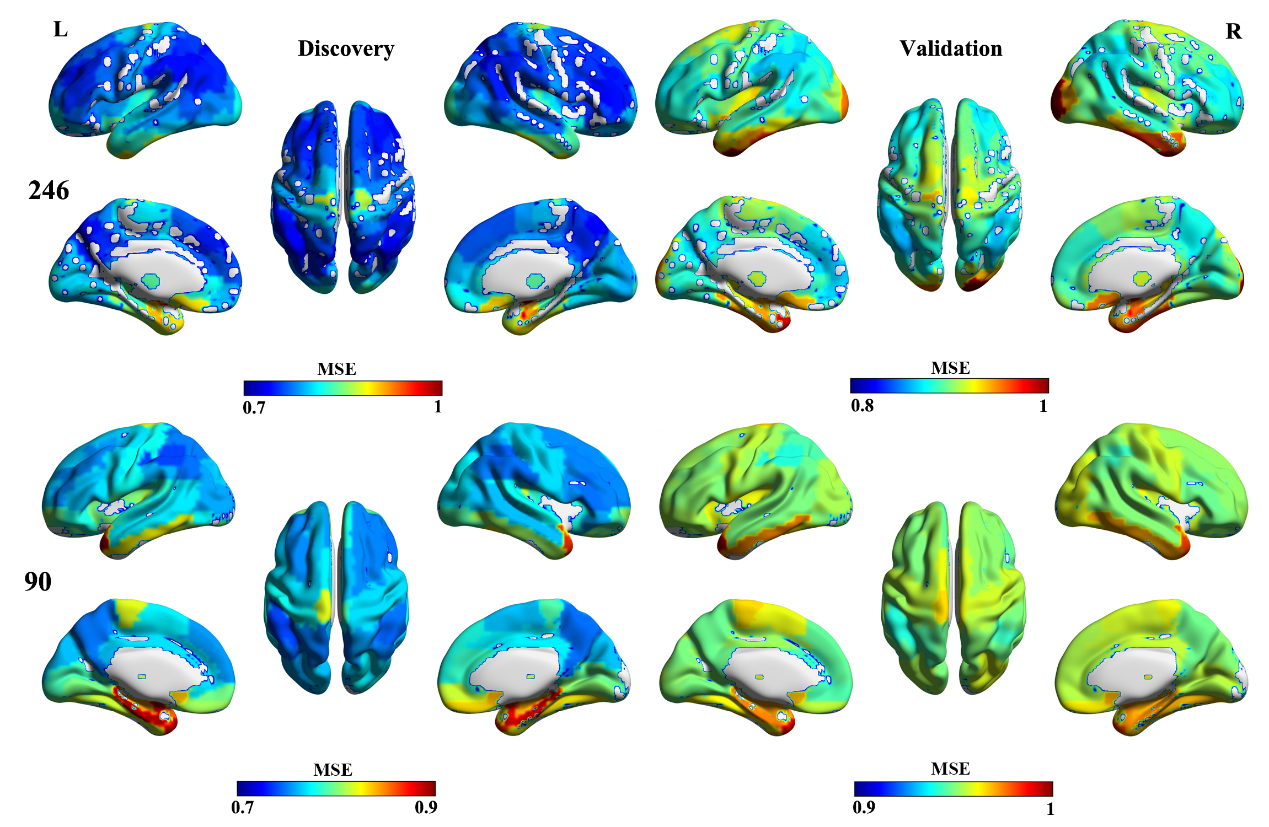


**Figure S4**. The overlap of (percentage) patients showing an extreme deviation in each brain region where regional extreme deviations are defined as |Z| > 2.6 (corresponding to p < 0.005).


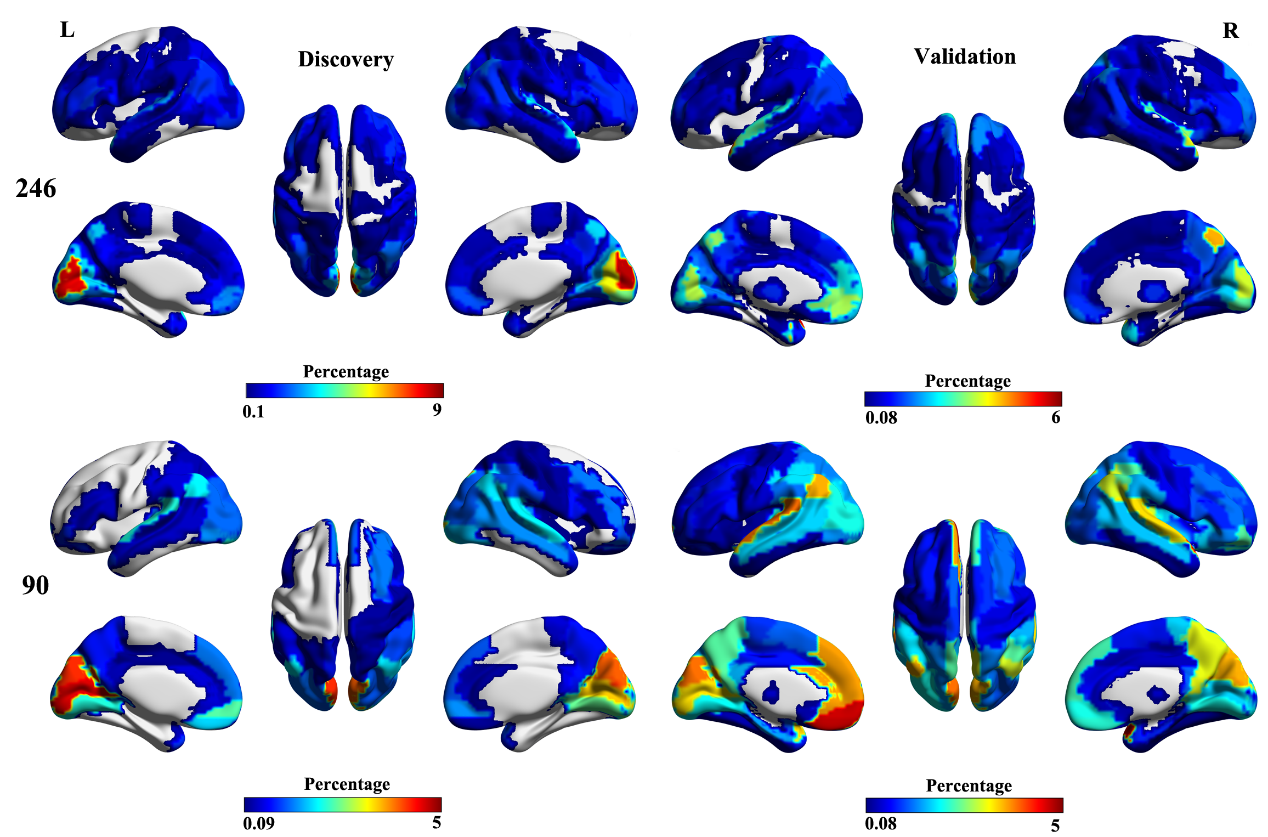


**Figure S5**. Generalizability error values of different numbers of differential factors in the discovery and validation datasets. Generalizability error values reach the minimum both for positive and negative factors, when the number of differential factors is 2 (red).


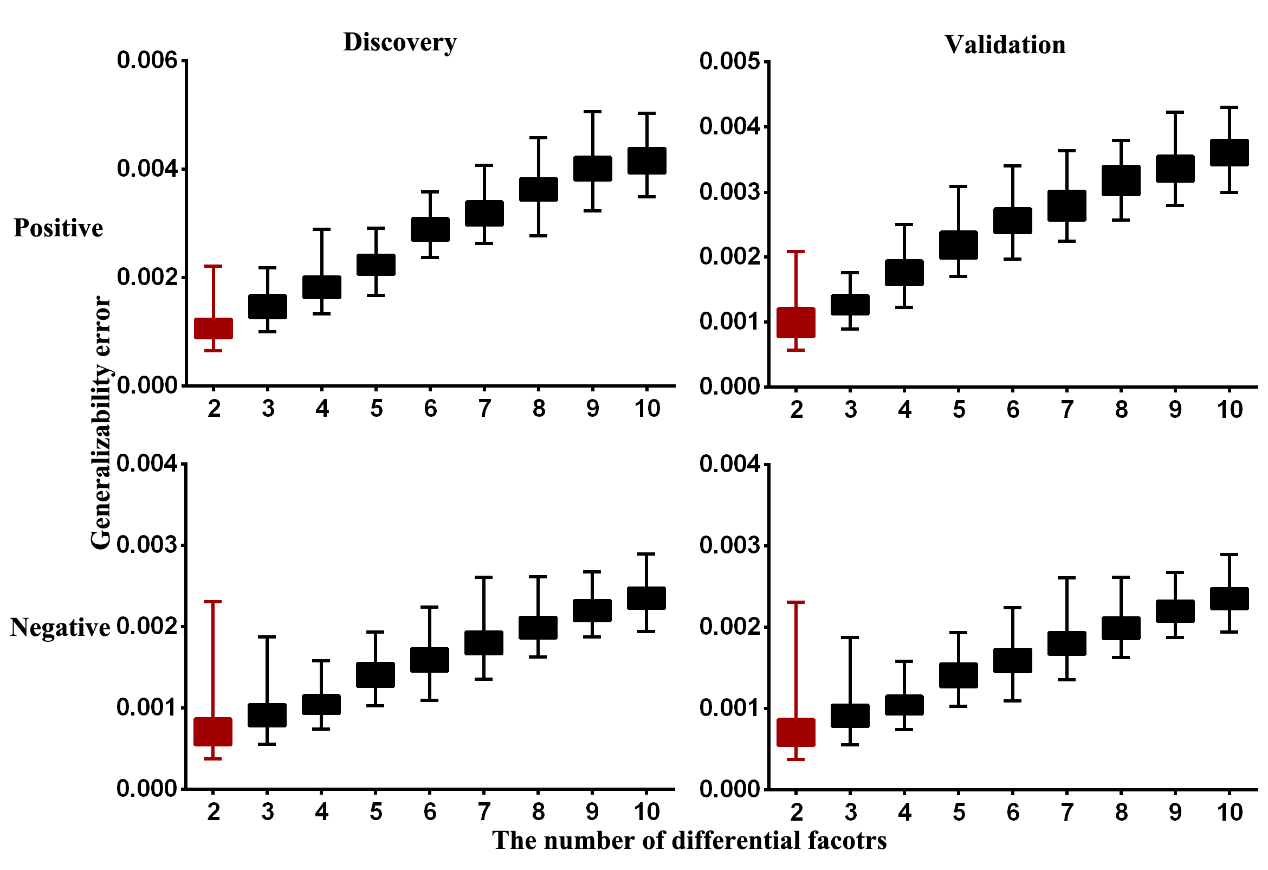


**Figure S6**. The most representative regions (the top 10% of 246 brain regions according to F values) of the identified differential factors and the corresponding factor composition (W) of patients in the validation dataset. Note, PF1, positive factor 1; PF2, positive factor 2; NF1, negative factor 1; NF2, negative factor 2.


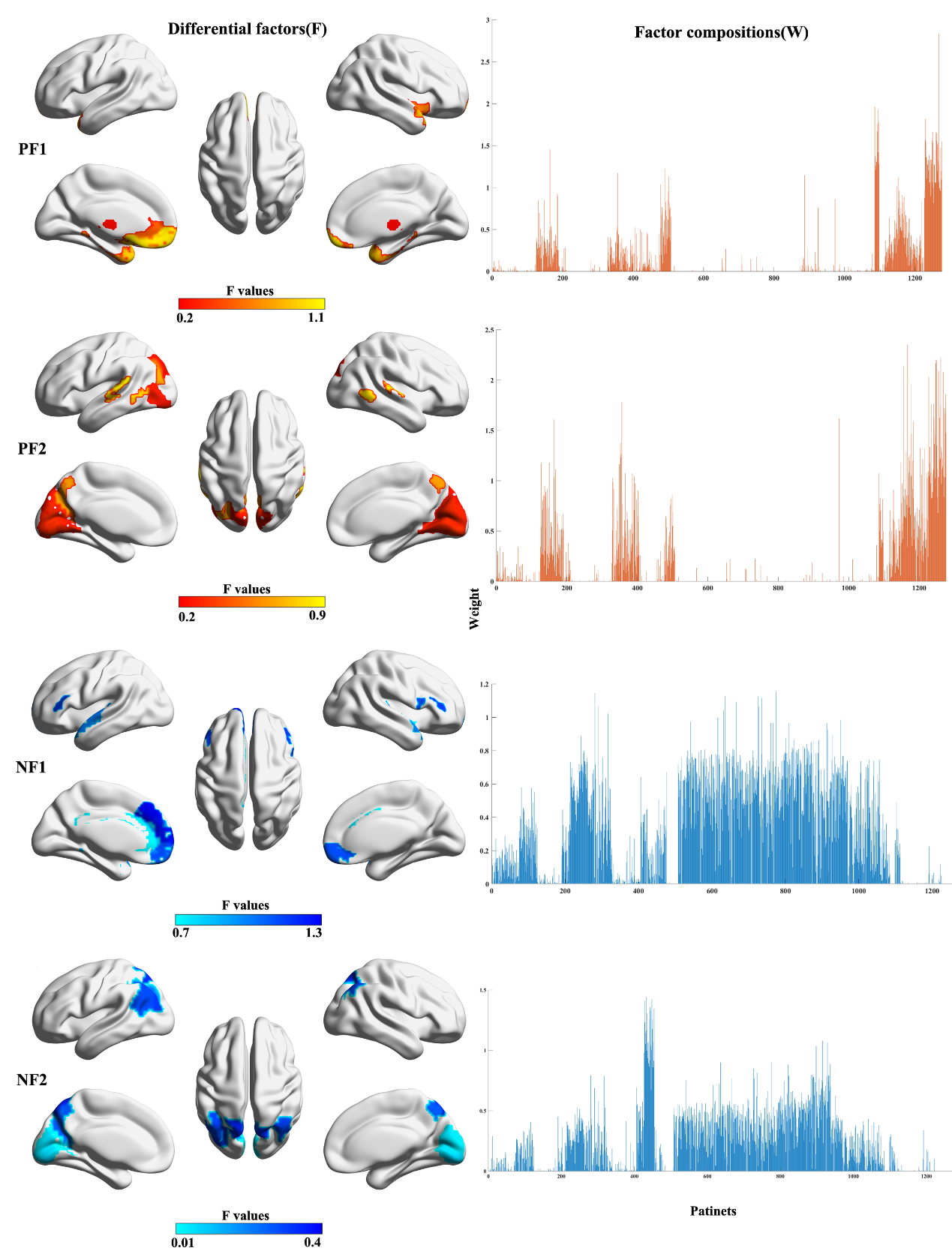


**Figure S7**. Pearson’s correlations between the identified differential factors using the discovery dataset and these using the validation dataset. All FDR-corrected *p* < 1.00e^-4^.


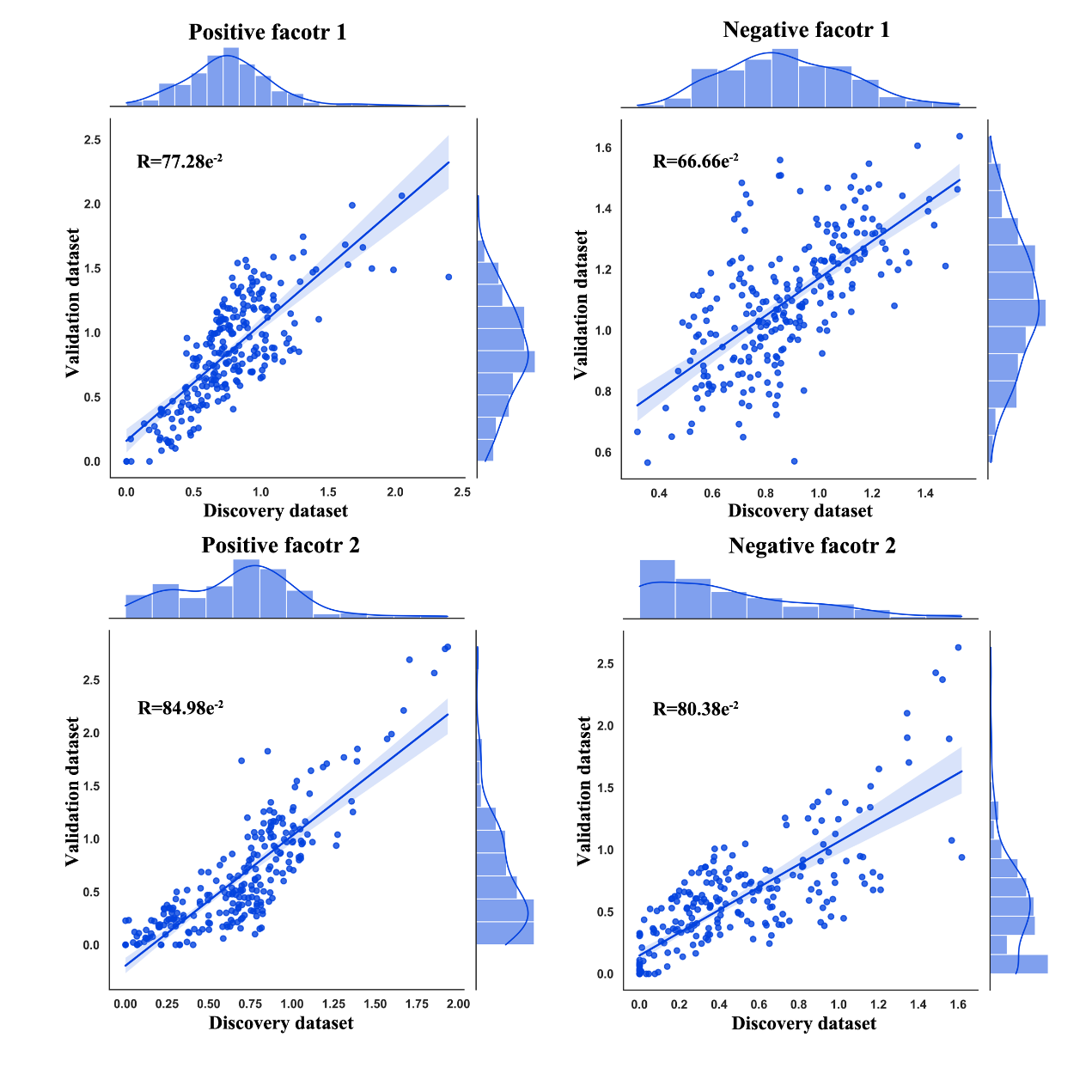


**Figure S8**. Group-level ALFF abnormalities of patients in the discovery and validation dataset.


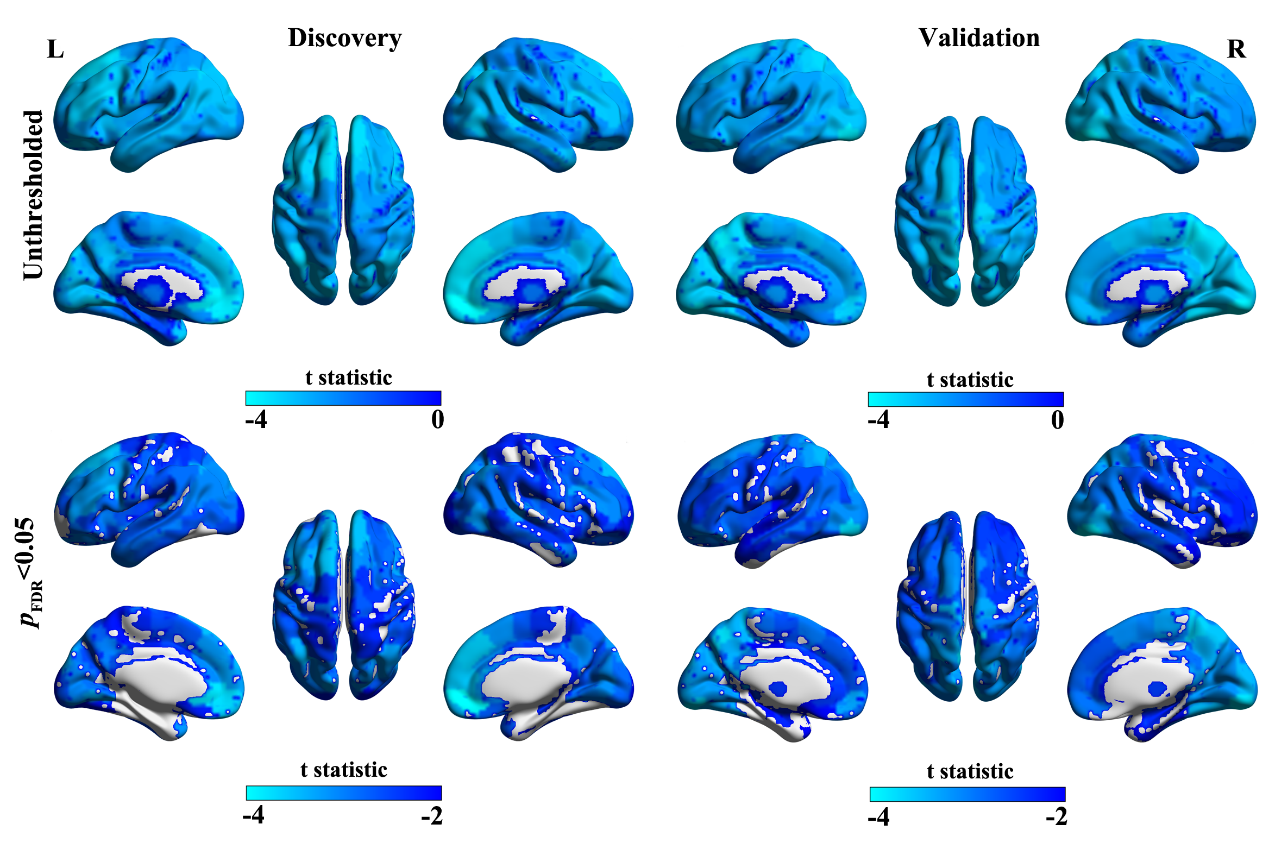


**Figure S9**. Impact of episodicity on the identified differential factors in the validation dataset. (A). Spatial correlations between identified differential factors using first-episode patients and those using recurrent patients. All FDR-corrected *p* < 1.00e^-4^. (B). Factor composition differences between recurrent and first-episode patients. Note, PF1, positive factor 1; PF2, positive factor 2; NF1, negative factor 1; NF2, negative factor 2.


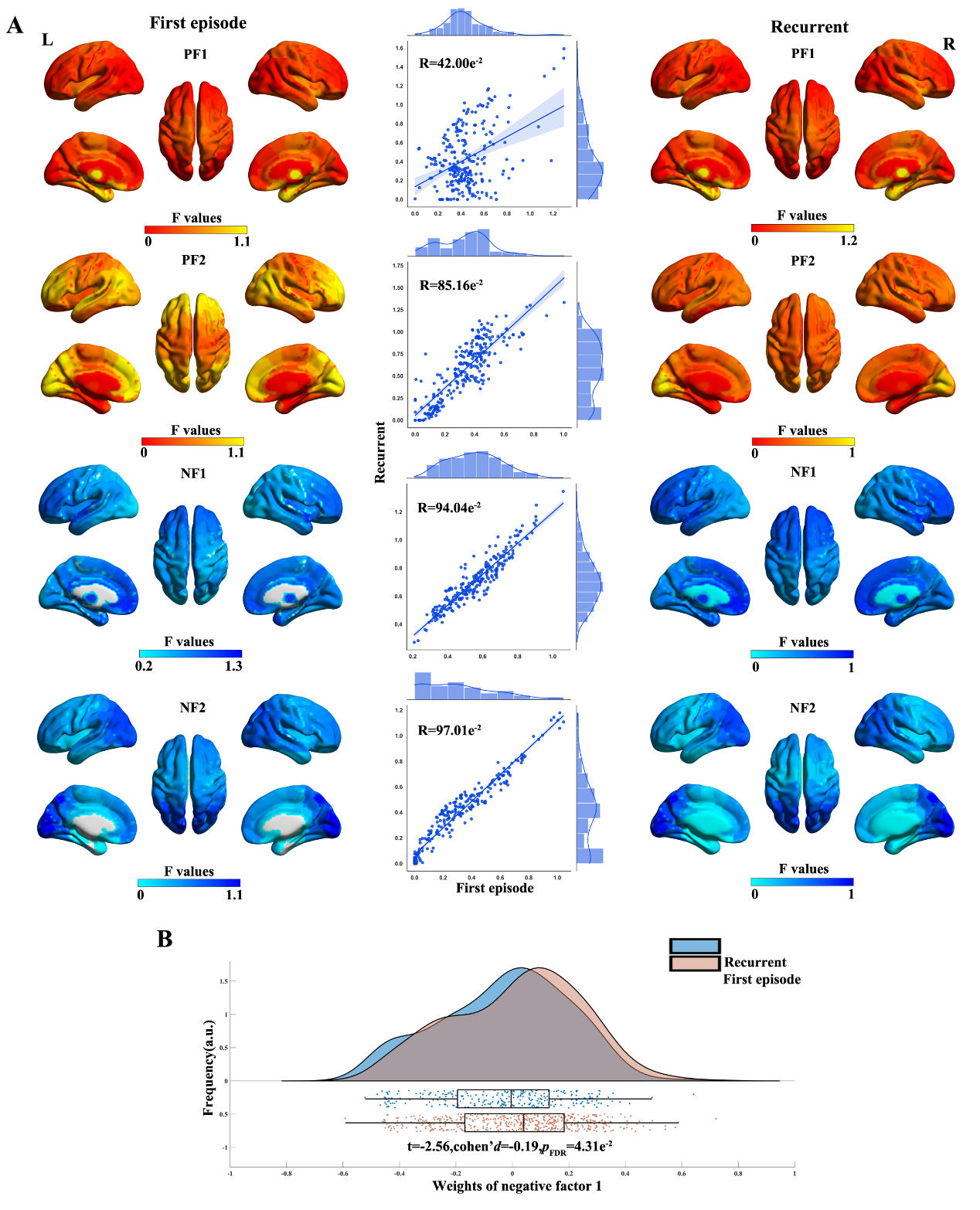


**Figure S10**. Impact of mediciation on the identified differential factors in the validation dataset. (A). Spatial correlations between the identified differential factors using first-episode patients and those using recurrent patients. All FDR-corrected *p* < 1.00e^-4^. (B). Factor composition differences between recurrent and first-episode patients. Note, PF1, positive factor 1; PF2, positive factor 2; NF1, negative factor 1; NF2, negative factor 2.


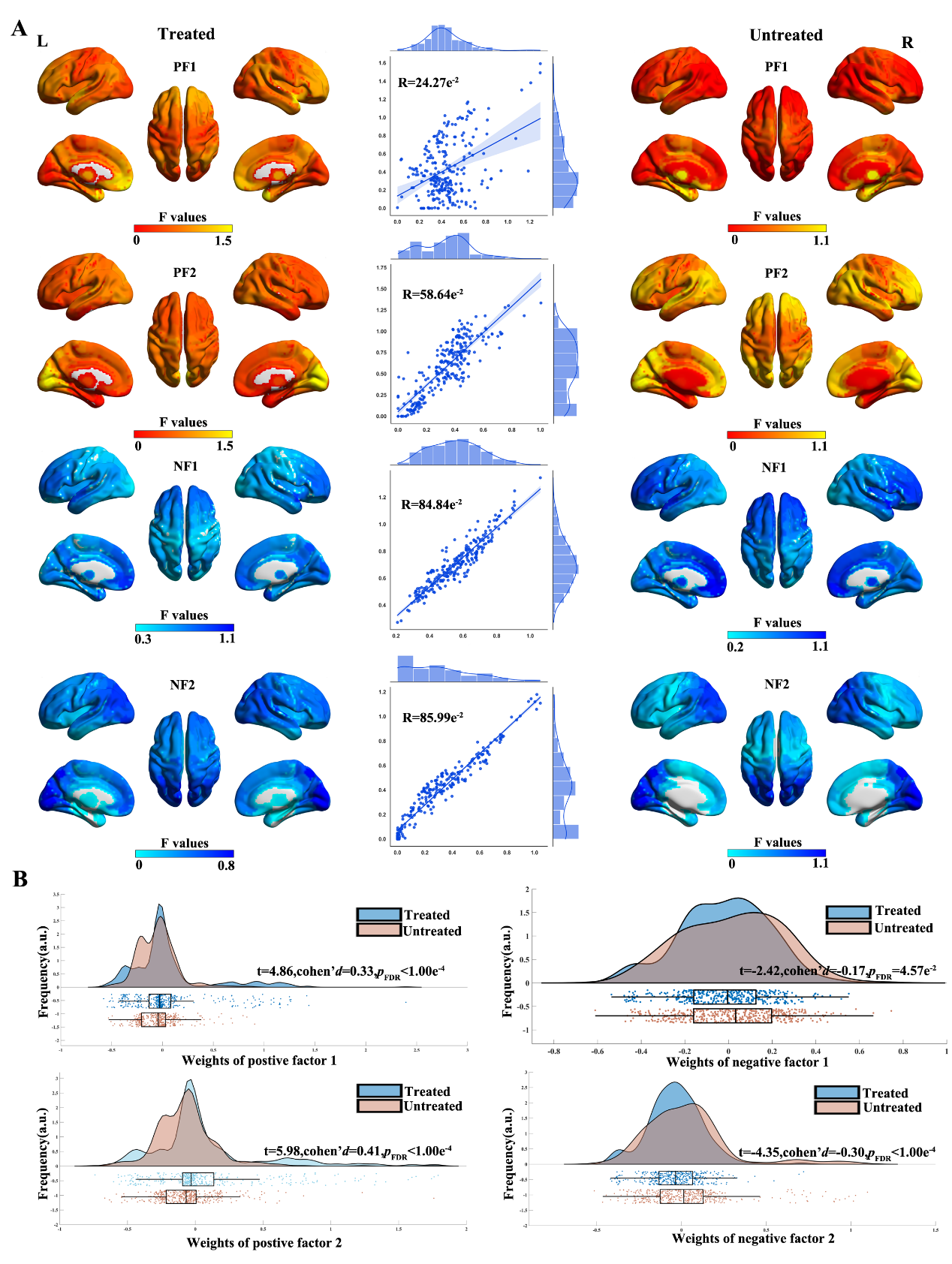


**Figure S11**. Correlations between the total scores of HAMD and factor compositions in the discovery dataset.


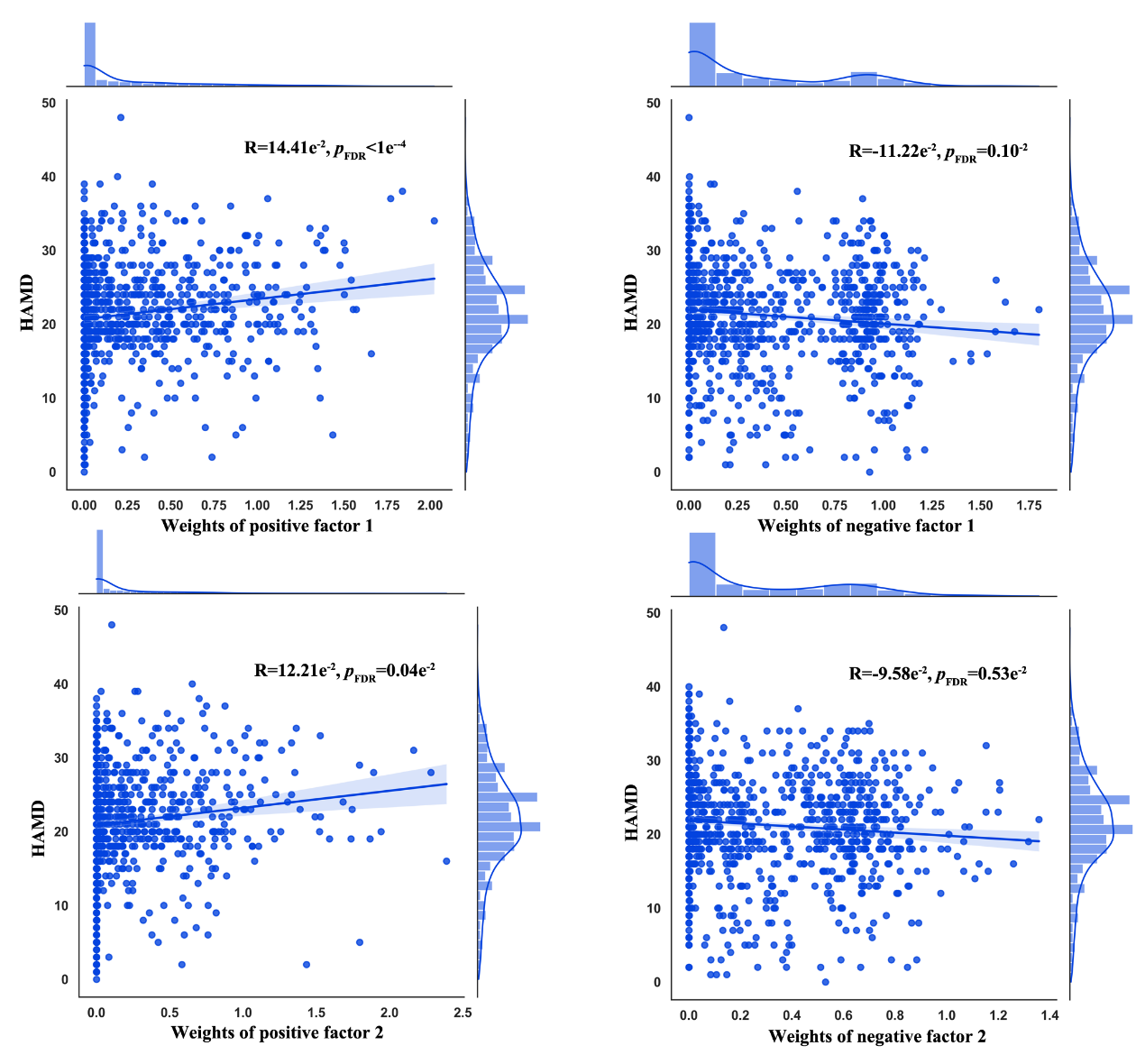


**Figure S12**. The association between the identified differential factors and normal structural covariance network (SC) in the validation dataset. (A). Pearson’s correlation coefficients between regional values and the normalized collective deformations of SC-informed values (the normalized collective deformations of structural neighbors) for each differential factor. (B). Th distributions of putative epicenters for each differential factor. Note, PF1, positive factor 1; PF2, positive factor 2; NF1, negative factor 1; NF2, negative factor 2.


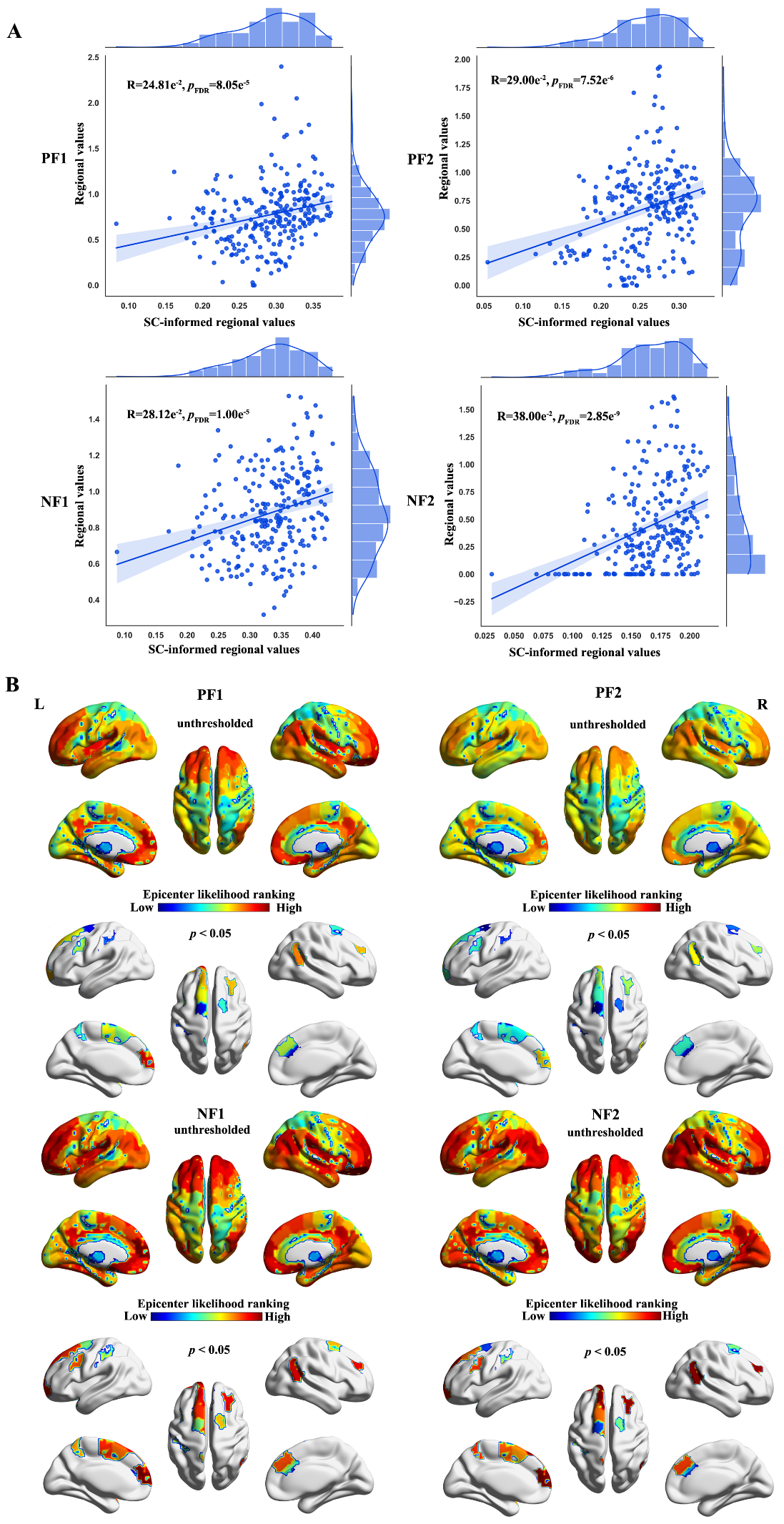


**Figure S13**. The association between neurotransmitter receptors/transporters and the identified differential factors in the validation dataset. (A). We construct four separate multilinear models of neurotransmitter receptors/transporters and each differential factor. The corresponding model goodness-of-fit (adjusted R^2^) is shown in the bar plot. (B). The permutation results of multilinear models. (C). The relative importance of the predictors for each multilinear model using Dominance analysis. The total dominance values, measuring the relative importance of the predictors, are shown. Note: PF1, positive factor 1; PF2, positive factor 2; NF1, negative factor 1; NF2, negative factor 2.


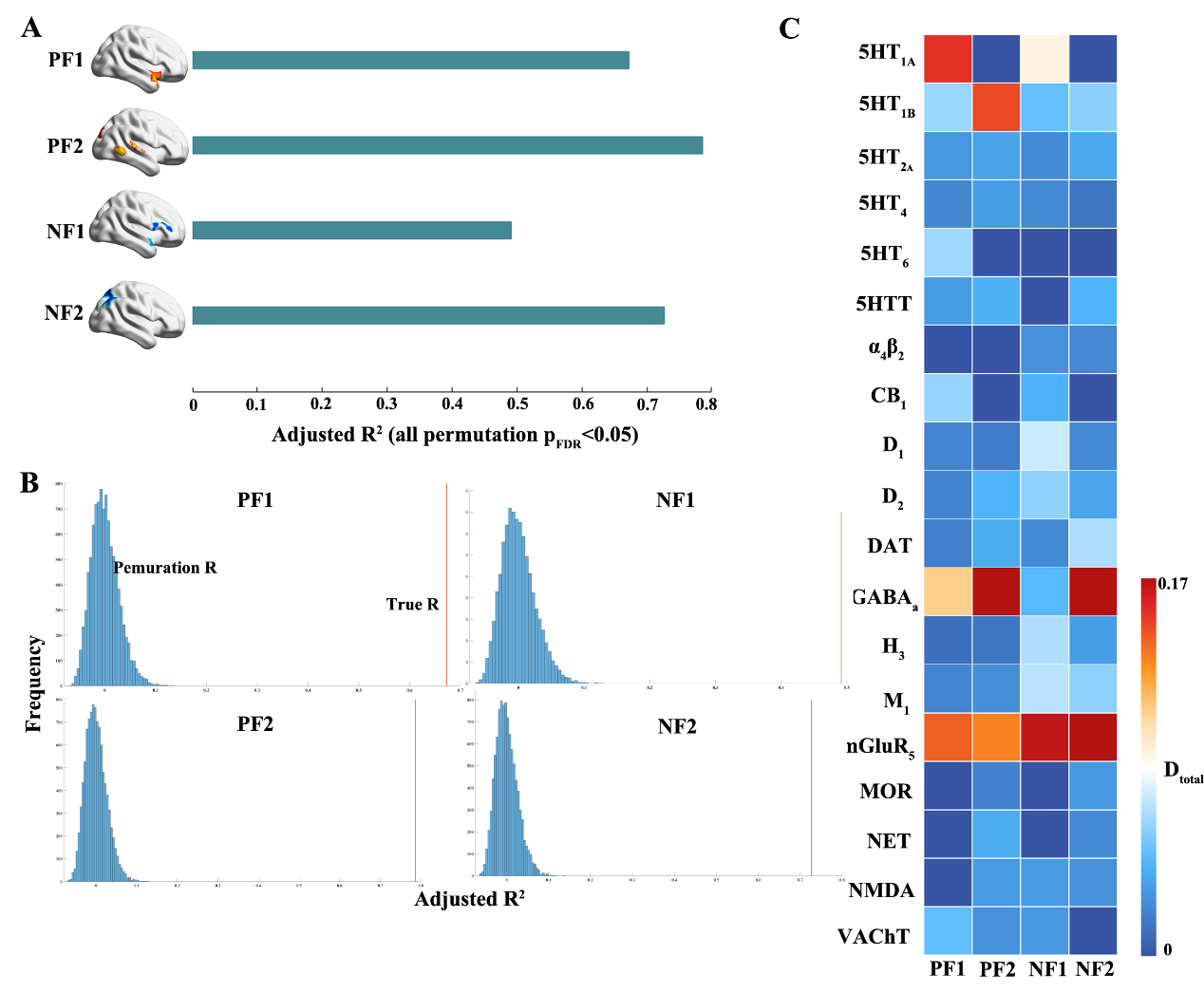


**Figure S14**. The association between differential factors and transcriptional profiles of inflammation-related genes in the validation dataset.


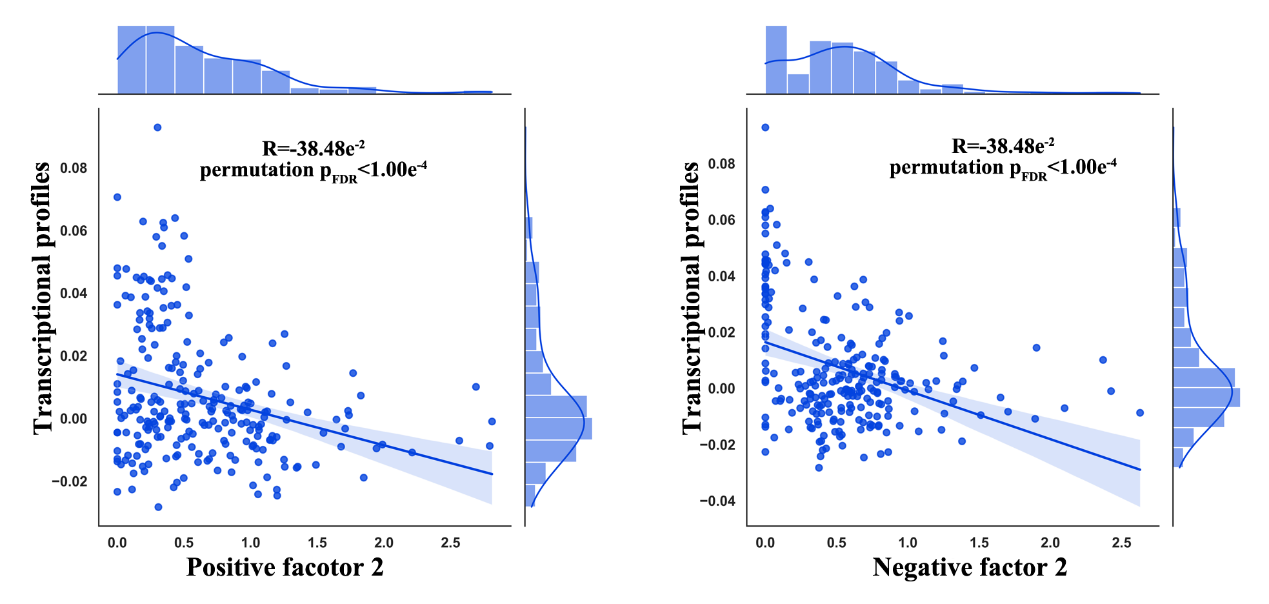


**Figure S15**. Subtyping results in the validation dataset. (A). BIC value for each number of subtypes. (B). Average factor compositions of each subtype. (C). ALFF abnormalities of each subtype relative to heathy controls. (D) Clinical characteristic differences among subtypes. Note, S1, subtype 1; S2 subtype 2; S3, subtype 3; PF1, positive factor 1; PF2, positive factor; NF1, negative factor 1; NF2, negative factor 2.


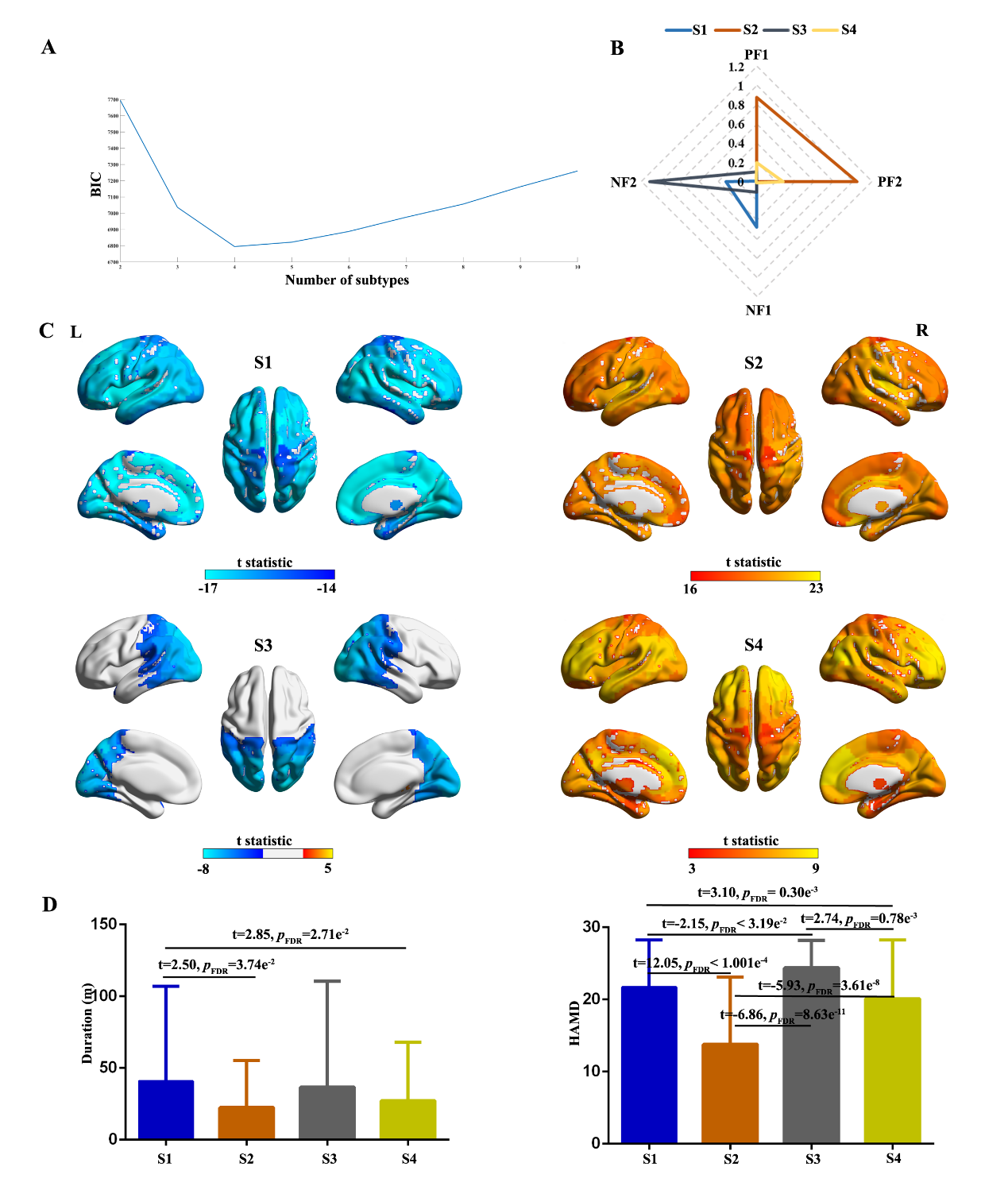


**Figure S16**. ALFF abnormities between subtypes using the discovery and validation datasets. The numbers represent the spatial correlation coefficients. Note, S1, subtype 1; S2, subtype 2; S3, subtype 3; S4, subtype 4.


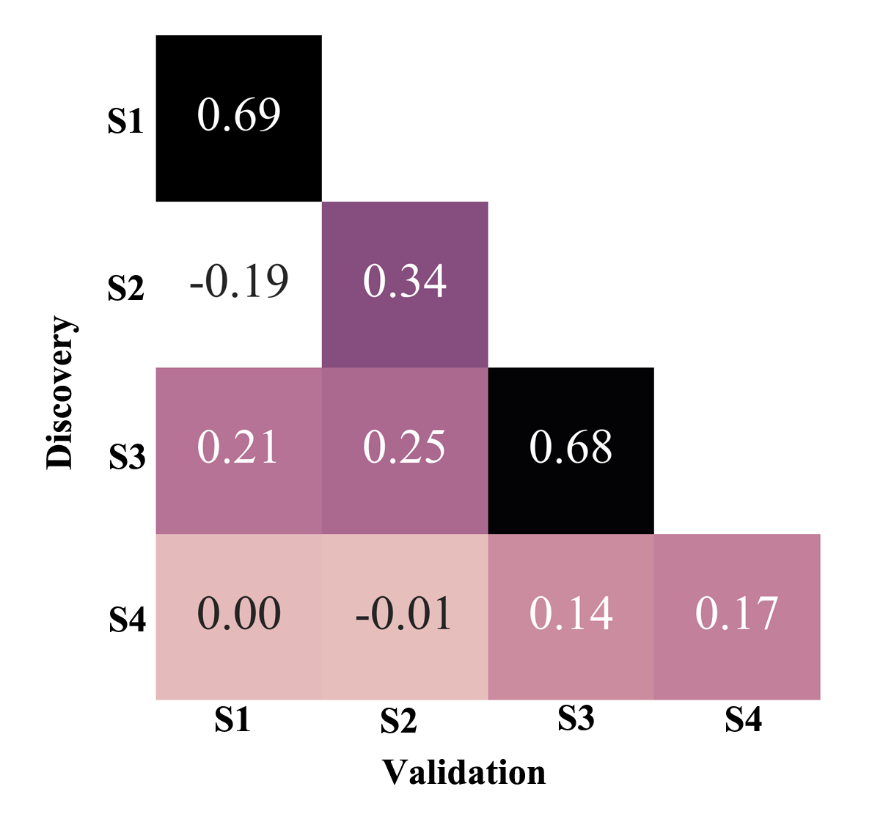


**Supplementary references**

1. Sun, X.Y., et al., *Mapping Neurophysiological Subtypes of Major Depressive Disorder Using Normative Models of the Functional Connectome.* Biological Psychiatry, 2023. **94**(12): p. 936-947.

2. Xia, M.R., et al., *Connectome gradient dysfunction in major depression and its association with gene expression profiles and treatment outcomes.* Molecular Psychiatry, 2022. **27**(3): p. 1384-1393.

3. Yan, C.G., et al., *Reduced default mode network functional connectivity in patients with recurrent major depressive disorder.* 2019. **116**(18): p. 9078-9083.

4. Chen, X., et al., *The DIRECT consortium and the REST-meta-MDD project: towards neuroimaging biomarkers of major depressive disorder.* Psychoradiology, 2022. **2**(1): p. 32-42.

5. Xia, M.R., et al., *Reproducibility of functional brain alterations in major depressive disorder: Evidence from a multisite resting-state functional MRI study with 1,434 individuals.* Neuroimage, 2019. **189**: p. 700-714.

6. Yan, C.G., et al., *Reduced default mode network functional connectivity in patients with recurrent major depressive disorder.* Proceedings of the National Academy of Sciences of the United States of America, 2019. **116**(18): p. 9078-9083.
